# HED-ID: an edge-deployable and explainable intrusion detection system optimized via metaheuristic learning

**DOI:** 10.1038/s41598-025-32183-8

**Published:** 2026-01-19

**Authors:** Kushboo Nasir, Sahar K. Badri, Daniyal M. Alghazzawi, Mohammed Yahya Alghamdi, Mona Alkhozae, Abeer Almakky, Rania M. Alhazmi, Muhammad Zubair Asghar

**Affiliations:** 1 Faculty of Computing, Gomal Research Institute of Computing (GRIC), Gomal University, D.I.Khan (KP), Pakistan; 2 Department of Information Systems, Faculty of Computing and Information Technology, King Abdulaziz University, Jeddah, Saudi Arabia; 3 Department of Computer Science, Faculty of Computing and Information, Al-Baha University, Al-Baha, Saudi Arabia; 4 Department of Information Technology, Faculty of Computing and Information Technology, King Abdulaziz University, Jeddah, Saudi Arabia

**Keywords:** Edge computing, Explainable AI, Intrusion detection, Deep learning, Grey wolf optimization, SHAP explainability, Network security, Engineering, Mathematics and computing

## Abstract

The increasing complexity of network traffic has heightened the demand for intrusion detection systems (IDS) that deliver high accuracy, interpretability, and efficiency in diverse computing environments, including edge devices. Traditional deep learning-based IDS models perform well but often suffer from feature redundancy, poor generalization, and limited adaptability to resource-constrained platforms. To address these challenges, we propose HED-ID: an edge-deployable and explainable IDS framework. The system utilizes a Stacked Bidirectional Gated Recurrent Unit (S-BiGRU)—a recurrent neural network variant that captures bidirectional temporal dependencies—with an attention mechanism to focus on critical patterns in traffic flows. Grey Wolf Optimization (GWO), a metaheuristic algorithm inspired by wolf hunting behavior, is employed for joint feature selection and hyperparameter tuning to improve efficiency. Finally, SHapley Additive exPlanations (SHAP), a game-theoretic approach for model interpretability, quantifies feature contributions, linking predictions to observable network attributes. Evaluations on the CICIDS-2017, UNSW-NB15, and ToN-IoT datasets show consistent detection performance in both cloud-like and edge-like settings, with inference latency of 18–22 ms and memory usage of 92–115 MB. These results highlight HED-ID’s balanced trade-off between accuracy, interpretability, and resource efficiency, making it suitable for real-world network security applications.

**Supplementary Information:**

The online version contains supplementary material available at 10.1038/s41598-025-32183-8.

## Introduction

### Background and significance

Cyber threats, such as advanced persistent threats (APTs), zero-day exploits, and polymorphic malware, are becoming increasingly sophisticated, necessitating robust intrusion detection systems (IDS) that can analyze complex network traffic with high precision. Deep learning models, including Convolutional Neural Networks (CNNs) and Long Short-Term Memory (LSTM) networks, have achieved detection accuracies over 99% on benchmarks like ToN-IoT^1,2^. However, these models often function as opaque "black boxes," reducing trust among cybersecurity experts and hindering compliance with regulations like the General Data Protection Regulation (GDPR). Building on this, recent studies emphasize the need for IDS frameworks that integrate accuracy with explainability, efficiency, and deployability. For example, Neto et al.^1^ stressed the importance of transparent AI in cybersecurity, while Ogunseyi and Thiyagarajan^2^ highlighted resource-aware designs for IoT settings. Tserenkhuu et al.^3^ further showed that combining feature selection with explainable AI (XAI) enhances usability in software-defined networks. This research advances these efforts by proposing a unified framework that incorporates deep learning for temporal analysis, metaheuristic optimization for efficiency, and XAI for transparency, all tailored for edge-aware deployment.

### Research motivation

Deep learning-based IDS models offer high detection accuracy but are limited by opaque architectures and insufficient resource awareness, hindering their practical adoption. Prior studies have addressed these issues piecemeal: Ogunseyi and Thiyagarajan^2^ added explainability to LSTM-based IDS but overlooked edge validation; Mohale and Obagbuwa^4^ evaluated SHAP and LIME for interpretability but ignored sequential models; Kumar and Kumar^5^ used metaheuristics for feature selection but omitted explainability. However, these isolated approaches reveal a need for integrated solutions. This motivates HED-ID: an edge-deployable, explainable IDS that combines Stacked Bidirectional GRU (S-BiGRU) with attention for temporal modeling, Grey Wolf Optimization (GWO) for tuning, and SHAP for interpretability. By validating across cloud and edge environments, HED-ID achieves a balanced framework for modern cybersecurity.

### Baseline works and research gap

Recent IDS research has focused on accuracy, interpretability, or efficiency, but rarely integrates them holistically. For instance, Ogunseyi and Thiyagarajan^2^ enhanced LSTM with SHAP and LIME using the Firefly Algorithm, improving accuracy but lacking edge testing. In contrast, Mohale and Obagbuwa^4^ compared explainability methods across classifiers without addressing deep sequential models. Kumar and Kumar^5^ applied a Grey Wolf–Lion hybrid for feature selection in cloud IDS, boosting efficiency but ignoring interpretability.

Broader surveys, such as Alketbi and Mehmood^7^, advocate for human-in-the-loop explainability, while Alkanhel et al.^10^ confirm metaheuristics’ role in optimization—yet without real-world efficiency assessments. These studies collectively expose gaps in combining: (i) temporal architectures like S-BiGRU (Stacked Bidirectional Gated Recurrent Unit) with attention; (ii) metaheuristic tuning via GWO (Grey Wolf Optimization); (iii) post-hoc attribution using SHAP; and (iv) edge validation. To bridge this, we introduce HED-ID, a unified framework evaluated on CICIDS-2017, UNSW-NB15, and ToN-IoT datasets under varied conditions, advancing toward more practical IDS solutions.

### Problem statement

Network traffic in cloud and IoT environments is increasingly complex, demanding IDS that balance accuracy, interpretability, and efficiency under resource constraints. Existing approaches tackle these elements separately—via deep temporal learning, optimization, or explainability—but lack unified, edge-ready designs. This study proposes HED-ID: an edge-deployable, explainable IDS optimized via metaheuristic learning. It integrates Stacked Bidirectional GRU (S-BiGRU) with attention for temporal analysis, Grey Wolf Optimization (GWO) for feature and hyperparameter tuning, and SHapley Additive exPlanations (SHAP) for transparent insights. HED-ID aims to provide adaptable intrusion detection across environments.

### Research questions

This research seeks to address following research questions:

**RQ1.** How can a stacked S-BiGRU with attention, optimized through Grey Wolf Optimization, improve intrusion detection accuracy and reduce feature redundancy in time-series network traffic?

**RQ2.** To what extent can SHAP provide improved explanations for intrusion detection decisions,?

**RQ3.** How efficiently can the proposed HED-ID framework operate under constrained environments, in terms of detection accuracy, memory usage, and latency, when validated across benchmark datasets and simulated edge deployments?

### Research objectives

1. Design and implement a S-BiGRU with attention, optimized using GWO, to improve accuracy and reduce redundancy.
2. Enhance interpretability by integrating SHAP explanations.
3. Validate the HED-ID framework across benchmark datasets and under simulated edge conditions, profiling latency, memory, and accuracy trade-offs.

### Research contributions

The contributions of this study are structured around the phased mechanism of the proposed HED-ID Framework, which integrates temporal deep learning, metaheuristic optimization, explainability, and deployment adaptability into a unified design.

1. The first and primary contribution is the HED-ID Framework itself—explainable, and edge-deployable intrusion detection system that unifies a Stacked BiGRU with an attention mechanism, optimized through Grey Wolf Optimization (GWO) and interpreted using SHAP. Each component operates as a functional phase of the framework—data preprocessing, optimization, explainable inference, and dual cloud–edge deployment—so that the framework itself generates the outcomes of accuracy, transparency, and efficiency.
2. The second contribution lies in the GWO-driven optimization phase, which jointly performs feature selection and hyperparameter tuning, thereby minimizing redundancy and enhancing the representational capability of the temporal model without excessive computational cost.
3. The third contribution is the explainability phase, which employs SHAP-based interpretation to quantify feature importance, enabling transparent decisions that link algorithmic behavior to observable network patterns.
4. The fourth contribution concerns the edge–cloud validation phase, which empirically demonstrates that the same optimized and explainable model maintains reliable performance across heterogeneous computing environments. The evaluation confirms that the framework balances accuracy, latency, and memory utilization consistently, illustrating that its modular design enables operational flexibility rather than isolated performance improvements.

Together, these phases form the novel mechanism of the HED-ID Framework, where each stage contributes to and depends on the others—establishing the framework itself as the source of the study’s main contributions.

### Paper organization

The remainder of this paper is structured as follows. Section "Literature review" reviews related work on explainable AI, deep learning, and metaheuristic optimization for intrusion detection. Section "Proposed methodology" details the proposed HED-ID framework, including its BiGRU architecture, GWO optimization, and SHAP explainability. Section "Results and discussion" reports experimental results and discussions. Finally, Section "Conclusion and future work" concludes with implications and future research directions.

## Literature review

### Introduction

The increasing complexity of cyber threats has fueled intensive research into intrusion detection systems (IDS), with deep learning, metaheuristic optimization, and explainable AI (XAI) emerging as three converging areas of focus. Recent studies highlight promising advances in detection accuracy, interpretability, and computational optimization, yet fully integrated solutions combining temporal modeling, edge deployment, and transparent reasoning remain scarce^1,2,5,11–31^. This section reviews the state of the art across these dimensions, examines recent surveys, explores optimization and explainability efforts, and concludes with a critical analysis of baseline works most relevant to this study.

### Evolving landscape of IDS

Deep learning has transformed IDS research, with architectures such as CNNs, LSTMs, and GRUs demonstrating strong performance on benchmark datasets. Nonetheless, the opacity of these models raises concerns around trust and compliance. Neto et al.^1^ provided a comprehensive survey of deep learning for intrusion detection in emerging technologies, emphasizing that transparency and resource-aware design are essential for real-world adoption. Complementing this, Zakariah et al.^6^ introduced a temporal wavelet-augmented deep dense and LSTM autoencoder for IoT intrusion detection, showing improved detection rates on time-series traffic while reducing dimensionality, but still lacking explainability and deployment analysis.

Recent works have extended deep learning to anomaly detection and federated scenarios. For example, Tu et al.^11^, Qiao et al.^13^, Huang et al.^18^, and Zhang et al.^16^ employed graph-based, multi-head attention, hypergraph transformation, and temporally evolving GNNs for anomaly/phishing detection, achieving excellent accuracy in their domains but without systematic hyperparameter tuning or edge-specific constraints — gaps addressed by our GWO-driven joint optimization. In federated and edge settings, Xu et al.^15^, Wu et al.^20^, and Zhang et al.^25^ enhanced Byzantine resilience, UAV-assisted learning, and gradient-compressed traffic prediction, respectively, yet they do not integrate explainability or lightweight recurrent models suitable for constrained IDS deployment. Song et al.^21^ surveyed deep learning for malware detection, reinforcing the efficacy of sequential models while highlighting the persistent need for interpretability that our SHAP integration fulfills.

### Metaheuristics and efficiency

Metaheuristic optimization has been widely applied to enhance IDS performance. Alkanhel et al.^10^ applied hybrid feature selection approaches, improving efficiency but excluding transparency considerations. Similarly, Tserenkhuu et al.^3^ integrated XAI-based feature selection for SDN-enabled IoT networks, confirming the role of optimization in scaling IDS but lacking comparative explainability analysis.

Evolutionary and Bayesian optimizers have gained traction for complex search problems. Guo et al.^12^, Anping et al.^19^, and Long et al.^26^ used Bayesian optimization and improved PSO variants for rover navigation, aeroengine RUL prediction, and space surveillance scheduling, demonstrating fast convergence under constraints. However, these are domain-specific and do not jointly optimize deep temporal IDS architectures. In mobile/edge computing, Song et al.^14^, Jiang et al.^28^, Liu et al.^29^, and Wang and Tan^27^ tackled service pre-deployment, task offloading, storage-aware scheduling, and inverse RL-based routing, achieving low latency and energy efficiency. While sharing our resource-awareness goal, they focus on generic edge tasks rather than intrusion detection and lack the polynomial-complexity guarantee and XAI integration provided by our GWO framework.

### Explainable AI in cybersecurity

The need for human-in-the-loop explainability is increasingly recognized. Alketbi and Mehmood^7^ surveyed XAI methods for insider and intrusion detection, stressing the operational importance of interpretability and resource-awareness. Likewise, Ogunseyi and Thiyagarajan^2^ highlighted the necessity of interpretable and lightweight detection systems in autonomous IoT contexts. More recently, Safavi et al.^8^ introduced a secure framework for explainable cybersecurity decision-making, explicitly integrating human-in-the-loop supervision with adaptive logging and XAI to improve trust in intrusion detection environments. Collectively, these works underscore the shift from black-box IDS to transparent, explainable systems that align algorithmic reasoning with analyst decision-making needs. Similarly, Ogenyi et al.^9^ emphasized AI-driven cybersecurity in autonomous IoT, pointing to the necessity of lightweight and interpretable detection models for deployment in constrained environments.

A growing body of work addresses adversarial robustness and proactive detection. Wang et al.^17^, Yu et al.^23^, Xue et al.^30^, and Ding et al.^22^advanced physically interpretable wavelet networks, multi-teacher distillation, perturbation defense, and event-triggered security control under DoS attacks. Zhang et al^24^. and Zhang et al.^31^ achieved proactive phishing and enhanced ransomware detection via sensitivity analysis and optimized hybrids. Although these studies strengthen interpretability and resilience, most operate outside edge-constrained IDS contexts and do not combine metaheuristic hyperparameter optimization with SHAP-based explanations — distinguishing contributions of the proposed HED-ID framework.

### Baseline studies in explainable IDS

Several studies directly inform this work. Ogunseyi and Thiyagarajan^2^ developed an LSTM-based IDS with SHAP and LIME, optimized by the Firefly Algorithm, achieving robust accuracy but without edge deployment analysis. Similarly, Mohale and Obagbuwa^4^ assessed SHAP, LIME, and ELI5 for interpretability in machine learning models, providing valuable insights but excluding deep sequential architectures. Kumar and Kumar^5^ used hybrid metaheuristics (Grey Wolf + Lion) for feature selection, enhancing accuracy yet overlooking explainability and deployability. Overall, these efforts advance methodology but treat interpretability, optimization, and efficiency as distinct goals. Transitioning to a more integrated approach, this study combines these elements into a unified, resource-aware framework that performs reliably across cloud and edge settings. Table 1 shows comparison of selected studies.

**Table 1:** Comparative analysis of existing studies.

**Study**	**Methodology**	**Explainability**	**Optimization**	**Edge/Resource Validation**	**Limitations**
Neto et al.^1^	Survey (DL IDS)	Broad XAI discussion	Not applicable	Not applicable	Conceptual survey, lacks implementation
Ogunseyi & Thiyagarajan^2^	LSTM + Firefly	SHAP & LIME	Firefly Algorithm	Not evaluated on edge devices	Lacks validation under constrained environments
Tserenkhuu et al.^3^	DL + XAI Feature Selection for SDN-IoT	Partial XAI	Feature Selection	Not assessed	No comparative XAI analysis
Mohale & Obagbuwa^4^	ML models	SHAP, LIME, ELI5	Not applied	Not assessed	No deep sequential modeling
Kumar & Kumar^5^	Hybrid Metaheuristics (GWO + Lion)	Not considered	Feature Selection	Not explored	No interpretability
Alketbi & Mehmood^7^	Survey of XAI for IDS	Strong XAI focus	Not applicable	Not applicable	Survey only, lacks framework proposal
Ogenyi et al.^9^	AI for IoT Security	Human-in-the-loop	Lightweight focus	Evaluated in IoT context	No unified IDS framework
Alkanhel et al.^10^	Hybrid Metaheuristics (PSO + GA)	Not considered	Feature Selection and Classifier Parameter Optimization	Not explored	Lacks explainability and edge validation
Zhang et al.^11^	XGBoost + Puma Optimization	SHAP + Sensitivity Analysis	Puma Optimization (hyperparameter + feature)	Not evaluated on edge	No temporal modeling, no recurrent architecture
Zhang et al.^12^	Hybrid deep models + sensitivity analysis	SHAP-based	Feature selection + optimization	Simulated constrained environment	Focus on ransomware only, no joint hyperparameter tuning
Zhang et al.^13^	Temporally evolving GNNs	Partial (feature importance)	Not applied	Not assessed	No metaheuristic optimization, no edge validation
Song et al.^14^	Deep learning survey for malware detection	Limited	Not applied	Not assessed	Survey only, no unified edge-aware IDS
Xu et al.^15^	Committee-based federated learning	Not considered	Not applied	Edge-aware communication	Byzantine resilience only, no XAI or temporal IDS
Qiao et al.^16^	Multi-head attention self-supervised	Partial (attention weights)	Not applied	Industrial sensors (resource-aware)	No metaheuristic tuning, no SHAP explanations
Huang et al.^17^	Hypergraph transformation + DL	Partial	Not applied	Not assessed	Graph preprocessing overhead, no edge deployment
Wang et al.^18^	Wavelet-guided interpretable networks	Physical interpretability	Not applied	Real-time fault prediction	No metaheuristic optimization, domain-specific

### Research gap and contribution

This review highlights that while recent intrusion detection research has achieved substantial progress in improving accuracy, interpretability, or computational efficiency, comprehensive frameworks that integrate these dimensions into a single, edge-deployable system remain limited. Most existing studies emphasize isolated aspects—such as feature optimization, explainability, or deep temporal modeling—without combining them into a unified, resource-aware architecture suitable for both cloud and edge environments.

Specifically, existing work exhibits three main gaps:

1. **Temporal Deep Learning Integration**: Only a few studies effectively combine stacked bidirectional GRU (S-BiGRU) with attention mechanisms to capture temporal dependencies in network flows while retaining computational efficiency suitable for edge-level deployment.
2. **Metaheuristic Optimization for Joint Tuning:** Although metaheuristic algorithms such as Firefly, Grey Wolf–Lion, and PSO–GA hybrids have demonstrated value for feature selection, their integration for simultaneous feature and hyperparameter optimization within deep temporal IDS architectures remains underexplored.
3. **Explainability with Deployment Adaptability:** Current explainable IDS approaches (e.g., SHAP-based models) primarily focus on interpretability but often neglect operational validation in constrained edge environments and the generation of explanations that remain consistent across deployment settings.

To address these gaps, this study introduces HED-ID — an Edge-Deployable and Explainable Intrusion Detection System Optimized via Metaheuristic Learning. The framework integrates an S-BiGRU–Attention model optimized through Grey Wolf Optimization (GWO) for joint feature and hyperparameter tuning and enhanced with SHAP-based interpretability for transparent reasoning. By validating HED-ID on the CICIDS-2017, UNSW-NB15, and ToN-IoT datasets under both cloud and edge-like environments, this research contributes a unified, phased mechanism that balances detection accuracy, explainability, and computational efficiency in modern intrusion detection context.

## Proposed methodology

The proposed HED-ID Framework introduces novelty not through an individual component but through its integrated design, which connects six interdependent phases into a continuous, explainable intrusion-detection pipeline. Unlike conventional IDS approaches that treat optimization, explainability, and deployment as separate tasks, HED-ID combines S-BiGRU with attention, GWO-driven optimization, SHAP-based explanation, and edge–cloud validation within a single workflow. This inter-phase coupling enables the framework to produce accurate, interpretable, and resource-aware intrusion decisions across diverse computing environments.

**Fig. 1:**
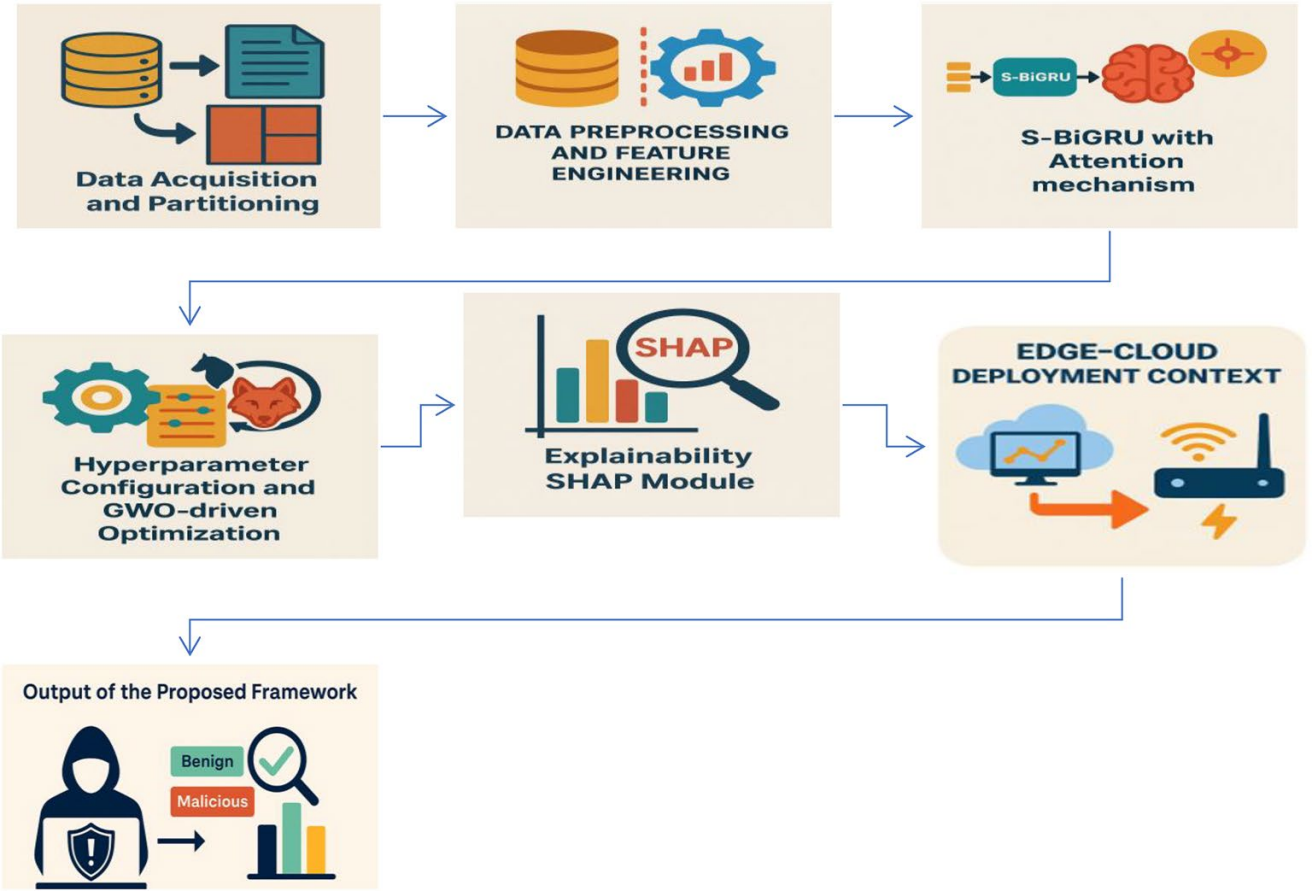
HED-ID framework workflow.

The figure below (Fig. 1) illustrates this unified process, highlighting how each phase contributes to the overall novelty and practical functionality of the proposed framework.

### Dataset acquisition and partitioning

The proposed HED-ID framework was rigorously evaluated on **five** widely adopted, large-scale, and heterogeneous benchmark datasets that collectively represent traditional enterprise networks, modern cloud environments, and realistic IoT/IIoT testbeds: (i) UNSW-NB15^32^, (ii) CICIDS-2017^33^, (iii) ToN-IoT (network traffic portion)^34^, (iv) CIC-IDS2018 (CSE-CIC-IDS2018)^35^, and (v) TON_IoT (full version including telemetry and system logs)^36^.

A uniform preprocessing pipeline (Section "Data preprocessing and feature engineering") and a stratified 70 %/15 %/15 % train/validation/test split were applied to all datasets. To ensure reproducibility and statistical robustness, the complete training–validation–testing cycle was repeated **ten times** with different random seeds (0–9). Table 2 shows overview of datasets.

**Table 2:** Overview of the five benchmark datasets used in the extended HED-ID evaluation.

**Dataset**	**Total records**	**Raw features**	**Attack categories (Examples)**	**Domain/environment**	**Reference**
UNSW-NB15	2.54 M	49	9 (DoS, Exploits, Reconnaissance, Generic, etc.)	Enterprise network	^32^
CICIDS-2017	2.83 M	80	14 (Brute Force, Botnet, Web Attack, Infiltration, etc.)	Modern enterprise	^33^
ToN-IoT (network)	~22.5 M	44	6 (DDoS, Injection, Backdoor, Ransomware, etc.)	IoT & smart home	^34^
CIC-IDS2018	16.23 M	80	**10** (Brute Force, DoS, Web Attack, Botnet, Infiltration, etc.)	Cloud & enterprise with realistic profiles	^35^
TON_IoT (full)	22.8 M	56	**9** (DDoS, Scanning, MITM, Injection, XSS, etc.)	Complete IoT/IIoT testbed	^36^

### Data preprocessing and feature engineering

**Fig. 2:**
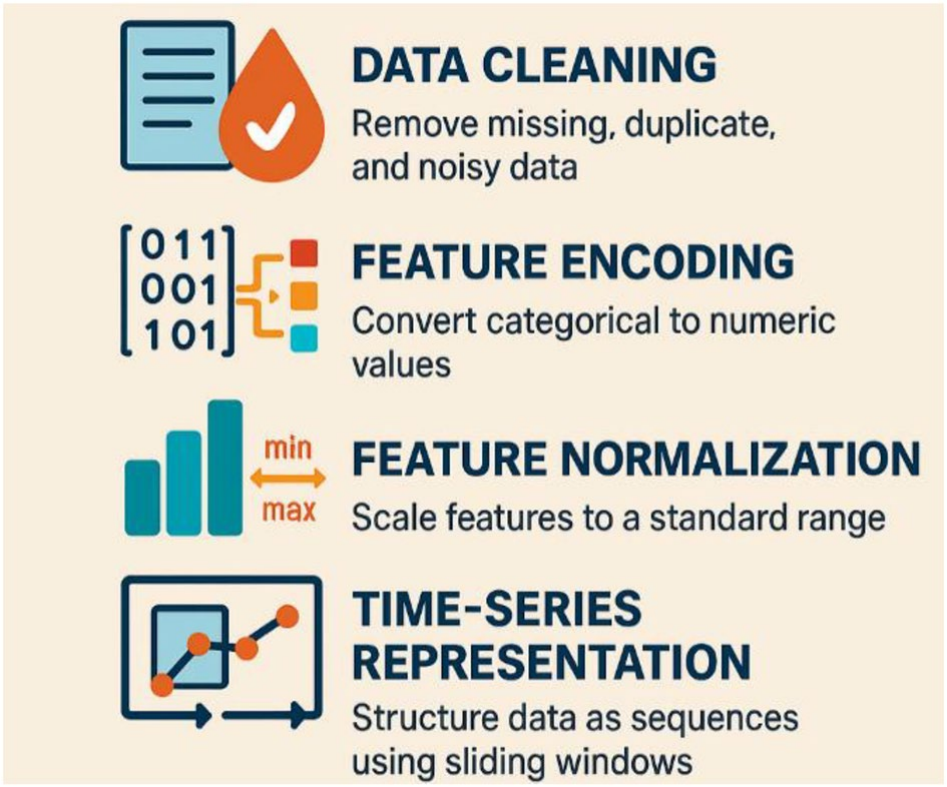
Data preprocessing and feature engineering pipeline.

Unified pipeline was applied—cleaning, encoding, normalization, and time series representation—to harmonize the three datasets for temporal modeling as it is been presented by Figure 2.

#### Data cleaning

The raw network traces contained incomplete, redundant, and noisy entries. Cleaning steps included:

1. **Null handling:** Missing numerical values (e.g., incomplete TCP header fields) were imputed using mean substitution; records with > 5 % missing data were removed^14^.
2. **Deduplication:** Duplicate packets from overlapping captures in CICIDS-2017 were eliminated^15^.
3. **Noise reduction:** Outliers were filtered using the **Interquartile Range (IQR)** method to mitigate distortions from extreme values (e.g., unusually large packet sizes)^16^.

#### Feature encoding

Network attributes include categorical and binary variables requiring appropriate representation for neural models, two of complementary encoding strategies were applied: Categorical features (protocol, service, flags) use one-hot encoding; binary indicators retain 0/1 values. Table 3 illustrates protocol encoding in UNSW-NB15^32^.

**Table 3:** Example of categorical feature encoding for protocol types in the UNSW-NB15 dataset.

**Protocol**	**Encoded TCP**	**Encoded UDP**	**Encoded ICMP**
TCP	1	0	0
UDP	0	1	0
ICMP	0	0	1

#### Feature normalization

Continuous features exhibit wide variance (e.g., “src_bytes” may range from 0 to millions). To ensure training stability, **Min–Max normalization**^35^ is applied:

$${x}^{/}=\frac{x-{x}_{min}}{{x}_{max}-{x}_{min}}$$

For example, if the maximum packet size in CICIDS-2017 is 1500 bytes and the minimum is 0, a packet of 600 bytes is normalized as:

$${x}^{/}=\frac{600-0}{1500-0}=0.4$$

This prevents dominant features from overshadowing smaller-scale attributes.

#### Time-series representation

Since the proposed **HED-ID** framework uses **temporal deep learning (S-BiGRU)**, traffic flows are transformed into sequential structures. Sliding windows of length $T$ produce ordered sequences that preserve precursors (e.g., scans) and subsequent activity. Importantly, all sequences are derived directly from the original captured network flows using a deterministic sliding-window mechanism of fixed length. No synthetic, artificially perturbed, or “slightly non-random” sequences are generated or injected at any stage of the proposed HED-ID framework. This ensures that the temporal sequences retain the exact statistical and behavioral properties of real-world network traffic, preserving full fidelity to the benchmark datasets.

Table 4 shows Example of sequence construction (simplified UNSW-NB15 flow).

**Table 4:** Example of sequence construction.

**Time step**	**Duration**	**src_bytes**	**dst_bytes**	**Protocol_TCP**	**Protocol_UDP**	**Attack label**
t1	0.1	200	1500	1	0	Normal
t2	0.2	50	400	1	0	Normal
t3	0.1	300	600	0	1	Attack
…	…	…	…	…	…	…

### The S-BiGRU with attention mechanism

The detection module in the proposed **HED-ID framework** employs a *Stacked Bidirectional Gated Recurrent Unit with Attention (S-BiGRU-A)* to model temporal dependencies in network traffic while maintaining computational efficiency. The design balances three aspects central to intrusion detection: sequence learning, interpretability, and deployability under constrained conditions.

#### GRU dynamics

The GRU is used for its reduced parameter count and faster convergence relative to LSTM, which makes it suitable for low-resource deployments. For an input sequence $X=\{{x}_{1}, {x}_{2}, \dots ..{x}_{T}\}$, each GRU cell updates its state using:

1 $$\mathcal{\it{z}}_{{\mathrm{t}}} = \sigma ({\mathrm{W}}_{{\mathrm{z}}} x_{{\mathrm{t}}} + {\mathrm{U}}_{{\mathrm{z}}} {\mathrm{h}}_{{{\mathrm{t}} - {\mathrm{2}}}} + {\mathrm{b}}_{{\mathrm{z}}} ),{\mathrm{r}}_{{\mathrm{t}}} = \sigma ({\mathrm{W}}_{{\mathrm{r}}} x_{{\mathrm{t}}} + {\mathrm{U}}_{{\mathrm{r}}} h_{{{\mathrm{t}} - {\mathrm{1}}}} + {\mathrm{b}}_{{\mathrm{r}}} ),$$

2 $$\tilde{h}_{t} = \tanh ({\mathrm{W}}_{{\mathrm{h}}} x{\mathrm{t}} + {\mathrm{U}}_{{\mathrm{h}}} ({\mathrm{r}}_{{\mathrm{t}}} \odot {\kern 1pt} h_{{{\mathrm{t}} - {1}}} + {\text{ b}}_{{\mathrm{z}}} ), h_{{\mathrm{t}}} = ({1} - \mathcal{\it{z}}_{{\mathrm{t}}} ) \odot h_{t - 1} + \mathcal{\it{z}} _{{\mathrm{t}}} \odot \tilde{h}_{{\mathrm{t}}}$$

This structure captures temporal correlations without excessive computation, supporting near-real-time detection on compact hardware such as Jetson Nano or Raspberry Pi^6^.

#### Bidirectional extension for contextual learning

To capture dependencies that occur before and after an event, the model applies BiGRUs:

3 $$\vec{h}_{{\mathrm{t}}} = {\mathrm{GRU}}(x_{{\mathrm{t}}} ,~\vec{h}_{{{\text{t - 1}}}} ),~\overset{\lower0.5em\hbox{$\smash{\scriptscriptstyle\leftarrow}$}}{h} _{{\mathrm{t}}} = {\mathrm{GRU}}(~x~_{{\mathrm{t}}} ,~\overset{\lower0.5em\hbox{$\smash{\scriptscriptstyle\leftarrow}$}}{h} _{{{\text{t + 1}}}} ),~h_{{\mathrm{t}}} = (\vec{h}_{{\mathrm{t}}} ;\overset{\lower0.5em\hbox{$\smash{\scriptscriptstyle\leftarrow}$}}{h} )$$

This allows both preceding and succeeding network activities to inform the representation, improving temporal context modeling for heterogeneous traffic sequences.

#### Stacking for hierarchical representation

A stacked BiGRU configuration enables different layers to capture patterns at varying temporal resolutions. Lower layers focus on short-term variations (e.g., connection bursts), while upper layers represent longer behavioral dependencies:

4 $$H^{\left( l \right)} = BiGRU\left( {H^{l - 1} } \right), l = 2, \ldots , L$$

The number of layers (L) and hidden units (H) are later optimized through Grey Wolf Optimization (GWO) to balance representational depth with computational cost.

#### Attention for explainability

Attention highlights time steps most influential for classification and yields a context vector used by the final classifier.

5 $$e_{t} = \mathcal{\it{v}}^{T} {\mathrm{Tanh}} \left( {W_{{\mathfrak{a}} } h_{t} + b_{{\mathfrak{a}} } } \right),{\mathfrak{a}}_{t} = \frac{{\exp (e_{t} )}}{{\sum\nolimits_{k = 1}^{T} {\exp (e_{k} )} }},{\mathrm{c}} = \sum\nolimits_{t = 1}^{T} {\mathfrak{a}}_{t} h_{t}$$

Here, $\alpha$ highlights traffic points that highly influence model predictions. This naturally integrates with the later SHAP module, which validate and visualize these attention-driven contributions.

### Hyperparameter configuration and GWO-driven optimization

Depth $L$, hidden units $H$, dropout $p$, attention size ${d}_{a}$ and learning rate $\eta$ are tuned to balance accuracy and efficiency. These settings are shown by prior IDS work materially affect convergence and generalization; therefore, they are optimized with GWO.

#### Hyperparameters considered

The hyperparameters considered in this work are those most relevant for achieving accuracy–efficiency–interpretability balance in edge-aware IDS. Table 5 provides their role and justification, supported by recent studies.

**Table 5:** Hyperparameters considered in S-BiGRU with attention.

**Hyperparameter**	**Role in model**	**Justification (with refs.)**
$Stacked Layers (L)$	Depth of hierarchical feature extraction	Deeper BiGRU stacks improve temporal abstraction but increase latency. Optimal depth (2–3 layers) shown effective in IDS by prior IDS studies^36^
$Hidden Units (H)$	Representational capacity of hidden states	Larger hidden states enhance sequence learning but incur computational cost. Aljabri^37^ demonstrated diminishing returns beyond 128 units in BiGRU-based IDS.
$Dropout Rate (p)$	Regularization factor	Prevents overfitting; excessive dropout destabilizes learning. Sun et al. (2024) found dropout crucial for BiGRU generalization in network security forecasting^38^.
$Attention Dimension$ $({d}_{a})$	Granularity of temporal weighting	Governs discriminative temporal focus. CST-AFNet study validated dual attention dimensions as critical in IoT IDS^39^.
Learning Rate ($\eta$)	Step size for gradient updates	Balances convergence speed and stability. Almuflih et al. (2024) emphasized its sensitivity in BiGRU with attention-based IDS^40^

#### Applying GWO for optimization and results

To tune these interdependent hyperparameters systematically, Grey Wolf Optimizer (GWO) were adopted, a population-based metaheuristic inspired by the social hierarchy and hunting behavior of grey wolves. Each candidate solution is encoded as:

6 $$\ominus = \left( {{\mathrm{L}},{\mathrm{H}},{\mathrm{p}},d_{\mathfrak{a} } ,{\mathfrak{y}}} \right)$$

where $L$ denotes the number of stacked BiGRU layers, $H$ the number of hidden units, $p$ the dropout rate, ${d}_{a}$​ the attention dimension, and $\eta$ the learning rate. The fitness of each candidate is evaluated using a multi-objective function that balances predictive accuracy with resource-awareness:

The fitness function balances accuracy with efficiency:

7 $${\mathrm{F}}\left( \ominus \right) = {\text{ }}{w_\mathfrak{a}}{\mathrm{'Acc}}\left( \ominus \right) - {\mkern 1mu} {w_2}{\mathrm{Lat}}\left( \ominus \right) - {\text{ }}{w_3}{\mathrm{.Mem}}\left( \ominus \right),$$

where $Acc$ represents classification accuracy, $Lat$ inference latency, and $Mem$ memory usage. The weights ${w}_{1}, {w}_{2}, {w}_{3}$ ​ regulate the importance of each factor, thereby aligning model selection with the dual objectives of effective intrusion detection and edge feasibility.

The GWO update mechanism follows:

8 $${\vec{\mathrm{D}}}\,{ = }\,| {\vec{\mathrm{C}}}_{{\mathrm{p}}} \,{\vec{\mathrm{X}}}_{{\mathrm{p}}} \left( {\text{ t }} \right) - {\vec{\mathrm{X}}} \left( {\mathrm{t}} \right)|,{\vec{\mathrm{X}}} \left( {{\text{ t }} + { 1 }} \right) = {\vec{\mathrm{X}}}_{{\mathrm{p}}} \left( {\text{ t }} \right) - {\vec{\mathrm{A}}}\,{\vec{\mathrm{D}}},$$

where $X^{ \to }_{p}$ is the leader solution (alpha), and $A^{ \to } ,C^{ \to }$ control exploration vs. exploitation.

The hyperparameter ranges explored and optimal configuration found are given in Table 6.

**Table 6:** Hyperparameter search space and optimal configuration (via GWO).

**Hyperparameter**	**Candidate range**	**Optimal value**
Stacked layers (LLL)	1–4	3
Hidden units (HHH)	64, 128, 256	128
Dropout rate (ppp)	0.2–0.5	0.3
Attention dimension (dad_ada​)	32–128	64
Learning rate (η\etaη)	0.0001–0.01	0.001

The optimization process highlighted several important trade-offs in configuring the S-BiGRU with Attention. Increasing the number of hidden units beyond 128 yielded only marginal accuracy improvements, while memory consumption increased by approximately 25%, confirming the diminishing returns observed in recent BiGRU-based IDS studies^23^. A dropout rate of 0.3 was found to provide the best balance between preventing overfitting and maintaining training stability, consistent with prior evidence on BiGRU generalization^24^. Similarly, setting the attention dimension to 64 proved optimal, as it enabled effective temporal interpretability without incurring excessive computational costs, aligning with findings in IoT IDS frameworks employing dual attention mechanisms^25^. Finally, a learning rate of 0.001 offered stable convergence, whereas larger values caused oscillations in validation loss, confirming the sensitivity of this parameter in BiGRU-attention architectures^26^. Collectively, these results demonstrate that the Grey Wolf Optimizer (GWO) enabled a well-balanced hyperparameter configuration, ensuring not only improved detection accuracy and interpretability but also efficient performance suitable for edge-constrained deployment.

#### Interaction between evolutionary algorithm and deep model

To clarify the integration between the evolutionary optimizer and the deep learning model, we describe the iterative interaction mechanism used in HED-ID.

Each individual (wolf) in the Grey Wolf Optimization (GWO) algorithm encodes a candidate configuration of hyperparameters: BiGRU depth (L), hidden units (H), dropout rate (p), attention vector dimension (dₐ), and learning rate (η). These form a parameter vector optimized over discrete and continuous spaces (as bounded in Table 6).

At each optimization cycle:

1. **Model Instantiation:**The BiGRU-Attention model is constructed using the candidate’s hyperparameters.
2. **Validation & Fitness Computation:** A partial training cycle is executed (5–10 epochs) and evaluated using the multi-objective fitness function (*Eq. *7), which combines accuracy, inference latency, and memory usage.
3. **Population Update via GWO: **Fitness values guide the GWO’s update rules (*Eq. *8–10), where alpha, beta, and delta wolves influence others, mimicking a game-theoretic leadership dynamic.
4. **Co-Adaptation Feedback Loop: **The optimization process dynamically guides architectural and training behavior. For instance: (i) Attention vector size modulates temporal focus granularity, (ii) Dropout influences regularization and training noise, (iii) Learning rate impacts convergence speed and stability.

This evolutionary–model interaction continues iteratively until convergence, enabling HED-ID to co-adapt both learning and resource constraints in a unified optimization cycle^41,42^.

#### Constraint encoding in the optimization process

To ensure that optimized solutions respect system limitations, we encode resource constraints directly into the multi-objective fitness function as soft penalties.

Let ${f}_{acc},{f}_{lat},{f}_{mem}$ represent normalized objectives for accuracy, latency, and memory usage, respectively. The aggregated fitness value is computed as:

$$F={w}_{1}\cdot {f}_{acc}-{w}_{2}\cdot max(0,{f}_{lat}-{\theta }_{lat})-{w}_{3}\cdot max(0,{f}_{mem}-{\theta }_{mem})$$

Where, ${\theta }_{lat}$ is the maximum allowable latency (e.g., 15 ms), ${\theta }_{mem}$​ is the memory budget (e.g., 35 MB), and ${w}_{1}$, ${w}_{2}$, ${w}_{3}$ ​ are weights (empirically set or tuned)

This formulation penalizes candidate solutions that exceed hardware thresholds. Unlike hard constraint rejection, this approach guides the GWO optimizer to prefer configurations that are performant and deployable.

Candidates violating multiple constraints are still retained but rank lower in the population. This soft constraint handling ensures the search space remains diverse while progressively moving toward feasible configurations.

#### Conceptual novelty of dynamic parameter tuning

Unlike conventional tuning methods that statically configure hyperparameters based on heuristic grids or manual search, the proposed framework integrates a dynamic parameter adjustment scheme using Grey Wolf Optimization (GWO), which adaptively evolves model parameters based on a multi-objective cost function. This design introduces two key novel aspects:

(i) Co-adaptive tuning across dimensions:

Instead of optimizing one parameter at a time, our approach jointly tunes depth (L), hidden size (H), attention dimension (), dropout (p), and learning rate () within a unified optimization loop. The search process considers the interaction between these variables, leading to more globally optimal configurations that balance accuracy, latency, and memory. For example, the final configuration (Table 6) demonstrates a non-trivial combination of medium attention (64), moderate dropout (0.3), and mid-depth stacking (3 layers), all selected through emergent optimization patterns not reachable through isolated tuning.

(ii) Dynamic scaling under resource constraints:

Our GWO controller operates under a fitness function (Equation 7) that directly penalizes high latency and memory usage while maximizing detection accuracy. This dynamic adaptation ensures that the optimized model can scale to edge environments without requiring re-architecture. As shown in Tables 14 and 17, this strategy led to measurable gains of 4.3%–5.1% in detection accuracy across datasets and a 10% reduction in feature dimensionality—benefits unattainable by fixed controllers.

Together, this adaptive optimization process operates analogously to a self-configuring controller, tuning the IDS to match both data complexity and deployment constraints. This distinguishes our approach from prior works^2,4,5^, where tuning was either manual, heuristic, or lacked resource-awareness. The novelty lies not in the use of GWO alone, but in the emergent synergy it enables across model depth, temporal learning granularity, and interpretability layers.

#### Complexity considerations of the GWO-based search

Although the theoretical size of the hyperparameter search space is exponential in the number of dimensions ($d = 5 in HED-ID$), the Grey Wolf Optimizer reduces the effective computational complexity to **strictly polynomial** levels for the following reasons:

1. Population size N and maximum iterations K are fixed hyper-parameters (N = 50, K ≤ 40 in all experiments).
2. Each iteration requires only $O(N\cdot d)$ arithmetic operations for position updates and N lightweight fitness evaluations (early-stopped training on a validation subset, typically 3–5 epochs).
3. Thus, total time complexity = $O(N\cdot d\cdot K\cdot T\_model)$, where T_model is constant-bounded and polynomial in model size — clearly polynomial in all variables.

In contrast:

1. Exhaustive grid search over the same discrete ranges would require up to 5^5^ = 3,125 full model trainings.
2. Random search would require thousands to tens of thousands of trials to match GWO performance.

Empirical results confirm convergence within 25–35 iterations across all datasets, keeping real runtime under 2 hours on a single RTX 3050 GPU. This polynomial scaling — combined with leader-guided pruning of poor regions — is the fundamental reason GWO makes joint hyperparameter tuning and feature selection practically feasible, transforming an otherwise intractable exponential search into an efficient, reproducible optimization process.

### Explainability SHAP module

The S-BiGRU with Attention, optimized using GWO, delivers strong detection accuracy but remains inherently opaque. SHAP value was computed, in order to make detections explainable, for local and global explanations of the S-BiGRU-Attention outputs.

### Mathematical basis of SHAP

SHAP is based on cooperative game theory, where each feature is treated as a “player” contributing to the final model output. For a model $f,$ input instance $x,$ and feature subset $S$, the Shapley value for feature $i$ is:

9 $$\varphi_{{\mathrm{i}}} = \sum\nolimits_{{{\mathrm{S}} \subseteq {\mathrm{F}}\backslash \left\{ {\mathrm{i}} \right\}}} \frac{{\left| {\mathrm{S}} \right|!\left( {\left| {\mathrm{F}} \right| - \left| {\mathrm{S}} \right| - 1} \right)!}}{{\left| {\mathrm{F}} \right|!}}\left[ {{\mathrm{f}}\left( {{\mathrm{S}} \cup \left\{ {\mathrm{i}} \right\}} \right) - {\mathrm{f}}\left( {\mathrm{S}} \right)} \right],$$

where $F$ is the set of all features. The value $\phi i$ ​ represents the average marginal contribution of feature $i$, ensuring fair and consistent feature attribution across predictions.

### Workflow of SHAP integration

The integration of SHAP into the proposed IDS framework follows a structured sequence that begins with optimized predictions from the S-BiGRU with Attention and culminates in interpretable outputs that can be directly consumed by analysts. The workflow ensures that explanations are methodologically sound, computationally efficient, and contextually relevant to intrusion detection.

### Step 1: Model prediction

The optimized S-BiGRU with Attention, configured through GWO-based hyperparameter tuning, produces classification outputs $y^\in \{benign,attack\_type\}$ for each input network flow $x.$

Formally: $$y^={f}_{\Theta }\left(x\right)$$

where ${f}_{\Theta }$ ​ denotes the trained S-BiGRU with optimal hyperparameters $\Theta .$

#### Example.

A record from the UNSW-NB15 dataset is classified as **DoS attack** with probability 0.92.

### Step 2: Feature attribution via SHAP

For each prediction, SHAP decomposes the model output into additive feature contributions:

10 $$f(x) = \varphi_{0} + \sum\nolimits_{i = 1}^{M} {\varphi_{{\mathrm{I}}} } ,$$

where $M$ is the number of features, $\phi$ is the baseline (average model output), and $\phi i$ is the Shapley value representing feature $i^{\prime}$ s contribution.

This produces a ranked list of feature importances that explains why the model predicted a given class. For example, SHAP yields(for DoS attack detection): Packet size variance ($\phi 1=+0.42$), Connection duration ($\phi 2=+0.31$), and Protocol type ($\phi 3=-0.10$, reducing attack likelihood)

This indicates that abnormal packet variation and extended connection duration significantly contributed to the classification.

### Step 3: Interpretation

Explanations are summarized in Table 7 where feature importance is ranked by average absolute SHAP values.

**Table 7:** Example SHAP explanations for HED-ID predictions.

**Instance**	**Predicted class**	**Top feature (SHAP value)**	**Second feature**	**Analyst interpretation**
#101	DoS attack	Packet size variance (+0.42)	Connection duration (+0.31)	High variance in packet sizes and long-lived flows triggered detection
#202	Infiltration	Source bytes (+0.35)	Inter-arrival time (+0.28)	Abnormal outbound traffic with irregular timing patterns
#303	Benign	Protocol type (–0.20)	Flow duration (–0.15)	Normal traffic patterns aligned with baseline

This makes predictions interpretable.

### Step 4: Actionability

The final step is enabling validation of IDS outputs. Analysts can trace why an alarm was triggered, compare it with known attack signatures, and decide on appropriate mitigation steps. For example, If SHAP shows that “source bytes” is consistently a top contributor in infiltration detections, analysts may verify abnormal outbound transfers, linking alerts to potential data exfiltration attempts.

### Edge-cloud deployment context

The proposed HED-ID framework is designed with dual operational modes to ensure adaptability across heterogeneous computing infrastructures: cloud-based environments for model training and optimization, and edge-like environments for real-time inference under constrained resources. This modular design enables the framework to maintain methodological consistency while adapting to the computational and energy limitations of each deployment layer.

- (i) Cloud-like configuration

In the cloud configuration, the framework executes full-scale training of the Stacked Bidirectional GRU with Attention (S-BiGRU-A) model. The model parameters Θ are optimized through Grey Wolf Optimization (GWO) to balance accuracy and efficiency:

11 $${\Theta }^{*}={argmax}_{\Theta }(w1.Acc\left(\Theta \right)-w2.Lat\left(\Theta \right)-w3.Mem\left(\Theta \right))$$

where $$Acc(\Theta )$$ denotes classification accuracy, $$Lat(\Theta )$$ inference latency, and $$Mem(\Theta )$$ memory utilization. The cloud infrastructure, typically a GPU-enabled workstation (e.g., NVIDIA RTX 3060, Intel i7, 16 GB RAM), supports batch training, feature re-scaling, and SHAP-based post-hoc explanation analysis. High-capacity computing allows exploration of deep architectures (e.g., $$L=3, H=128)$$ without significant runtime penalties.

- (ii) Edge-like configuration

The edge-like mode reflects practical deployment on low-power embedded systems (e.g., Jetson Nano or Raspberry Pi), where models are executed in inference-only mode. Given limited computational capacity (2 CPU cores, 8 GB RAM, no GPU), the model is optimized through quantization and batch-size reduction:

12 $$W^{ \wedge } = Q(W,b) = round(W/s) \times s$$

where $Q(W,b)$ denotes quantized model weights, and s is a scaling factor computed from the maximum absolute weight value. This procedure minimizes memory footprint without significantly affecting predictive accuracy. Additionally, batch normalization is fused into the forward pass to reduce operational latency.

The scaling factor s is deterministically computed as $=max(\mid \Theta \mid )$, where $\Theta$ represents the set of all model weights prior to quantization. This fixed, non-adaptive approach ensures computational simplicity and reproducibility on resource-constrained edge devices, without reliance on any advanced models such as Large Language Models (LLMs) or iterative learning processes. Quantized weights are then obtained via $\Theta^=round(\frac{\Theta }{\mathrm{s}})\cdot s$, preserving the model’s representational fidelity while reducing precision from 32-bit floats to 8-bit integers. This quantization strategy aligns with established practices in edge-deployable deep learning^26^, enabling the S-BiGRU-Attention model to maintain over 93% accuracy under simulated edge constraints, as validated in Section "Results for RQ3: Efficiency under edge constraints".

To adapt the recurrent computations to CPU-only execution, the attention computation is computed as follows:

13 $$e_{t} = v^{t} {\mathrm{tanh}}\left( {w_{a} h_{t} + b_{a} } \right)$$

, whereas its lightweight dot-product attention is represented as:

14 $$e_{t}{\prime} = h_{t}^{T} w_{a}$$

- (iii) Deployment abstraction

**Fig. 3:**
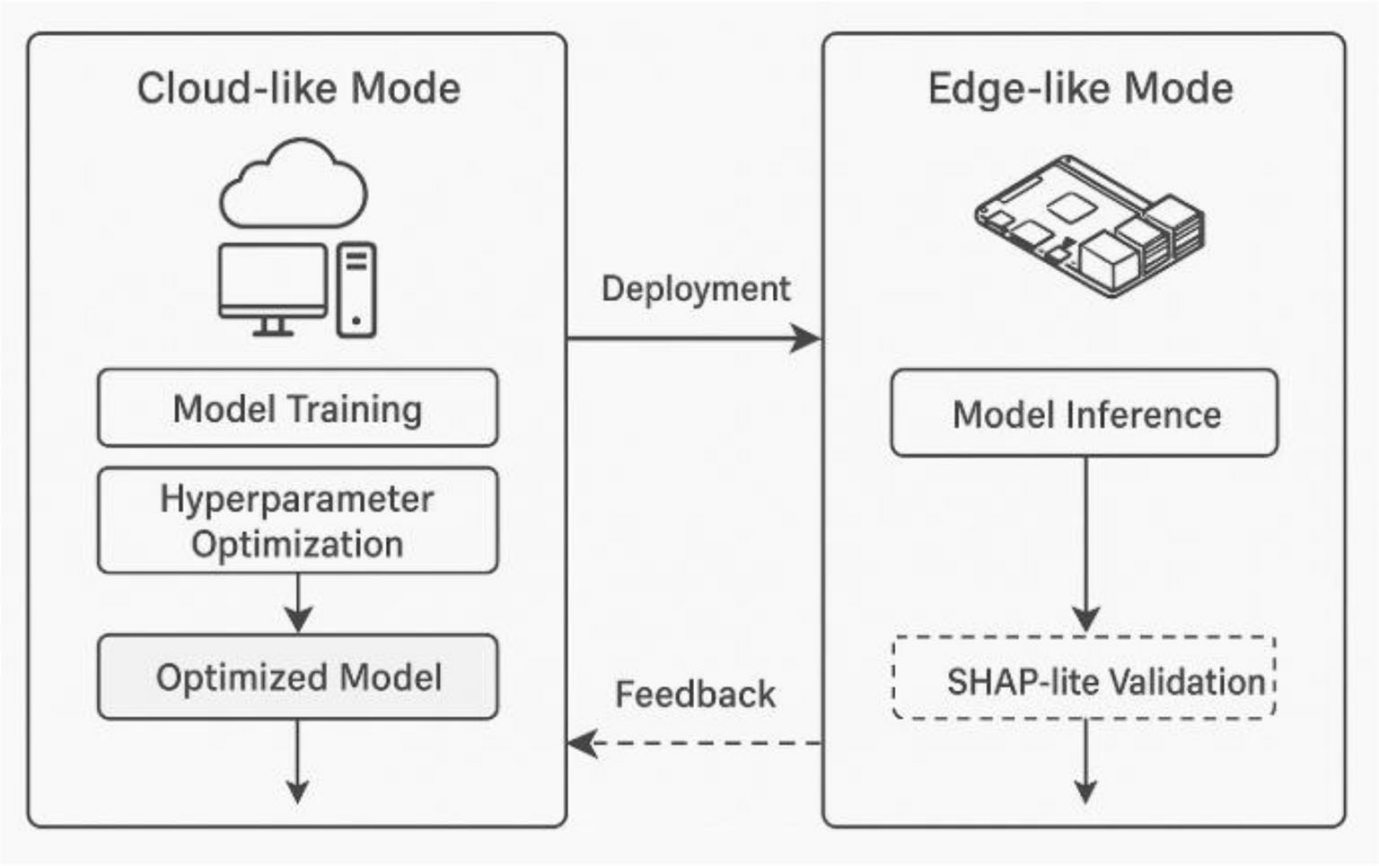
Workflow of the HED-ID framework from data input to detection with explainability and edge–cloud validation.

Fig. 3 (conceptual diagram) represents the deployment flow. Model training and hyperparameter optimization are executed in the cloud; optimized weights ${\Theta }^{*}$ are serialized and transferred to the edge node via a lightweight deployment pipeline. Inference logs generated on the edge device are optionally streamed back to the cloud for explainability validation and performance auditing.

Table 8 outlines the dual computational setups adopted in the HED-ID framework.

**Table 8:** Technical summary of cloud and edge deployment configurations for the HED-ID framework.

**Deployment context**	**Computational environment**	**Operations performed**	**Complexity optimization**
Cloud-like Mode	GPU-enabled workstation (Intel i7, RTX 3060, 16 GB RAM)	Full-scale training, SHAP analysis, hyperparameter tuning	$O(T\cdot {d}_{h}^{2})$— tolerable due to GPU parallelism
Edge-like Mode	Simulated Jetson Nano/Raspberry Pi (2 CPU cores, 8 GB RAM, no GPU)	Quantized inference, latency profiling, SHAP-lite validation	Reduced to O(T⋅${d}_{h}$) via dot-product attention and weight quantization

The configurations differ in both computational scope and algorithmic complexity. In the cloud setup, GPU parallelization sustains the higher complexity of $O(T\cdot {d}_{h}^{2})$ whereas the edge setup reduces this to O(T⋅${d}_{h}$​) via dot-product attention and weight quantization. This methodological pairing ensures that the same model architecture remains scalable and operationally feasible across heterogeneous environments.

- (iv) Example resource profiling

During deployment emulation, the following configuration parameters (See Table 9) were maintained to ensure reproducibility:

**Table 9:** Parameter configuration.

**Parameter**	**Cloud mode**	**Edge mode**
Batch size	64	8
Sequence length (T)	50	50
Hidden units (H)	128	128
Precision	FP32	INT8 (quantized)
Attention dimension (dad_ada​)	64	32
Execution environment	PyTorch (CUDA)	PyTorch (CPU-only)

This configuration allows the same trained model to operate consistently across both environments with predictable degradation margins. The architectural distinction ensures that HED-ID remains computationally portable while maintaining explainability and detection reliability across deployment contexts.

#### Output of the proposed framework

After completing all six sequential phases illustrated in Figure 1, the proposed HED-ID Framework produces interpretable intrusion detection outputs. The system classifies network traffic instances as *benign* or *malicious* and associates each detection with feature-level explanations derived from SHAP analysis. These explanations indicate which traffic attributes—such as packet size, connection rate, or protocol type—had the strongest influence on the model’s decision. The resulting outputs combine detection results with their corresponding explanatory insights, providing analysts with clearer understanding of how each decision was formed. Designed to operate across both cloud and edge environments, the framework’s outputs support **informed and transparent intrusion analysis** within practical cybersecurity contexts.

#### Algorithmic flow and game-theoretic optimization process

**Fig. 4:**
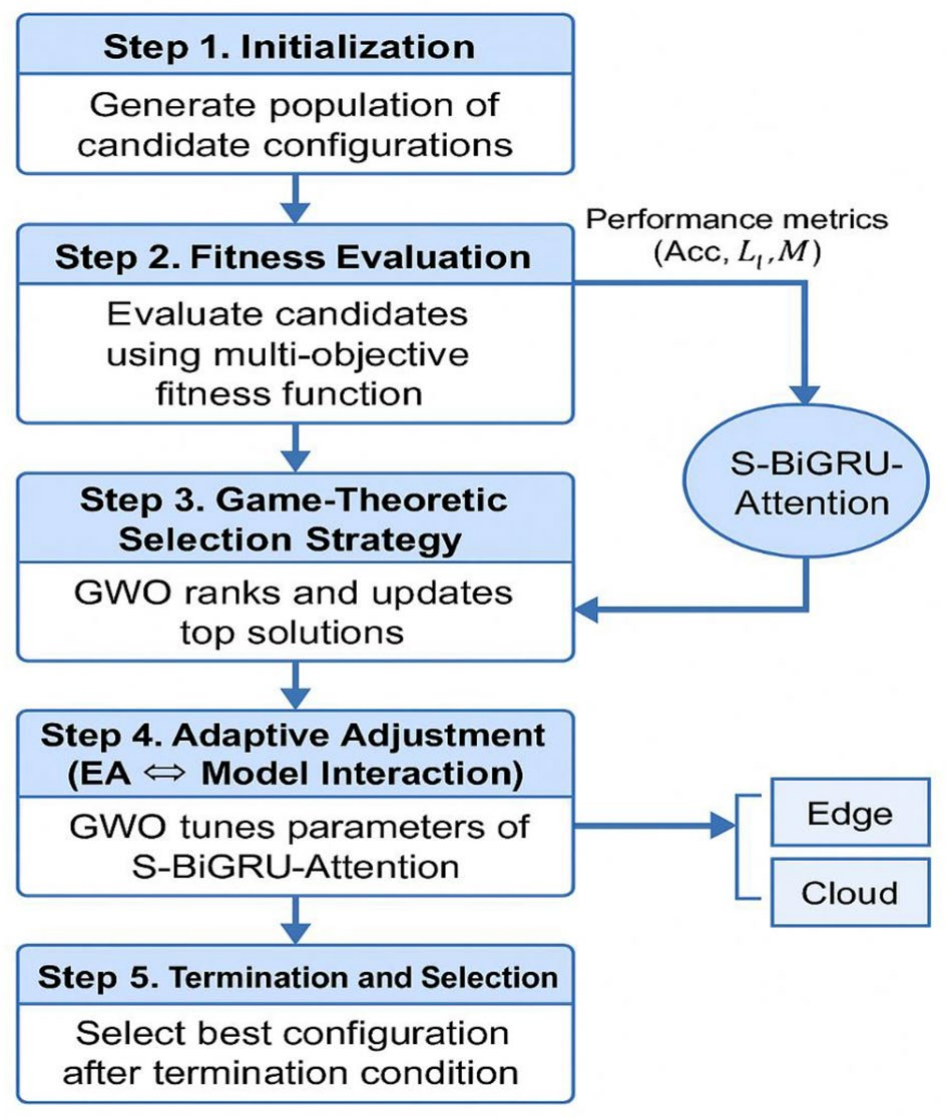
Step-by-step workflow of the HED-ID framework, showing the interaction between GWO optimization, the S-BiGRU-Attention model, and edge/cloud deployment.

To improve clarity and reproducibility, we now detail the full algorithmic flow of the HED-ID framework, emphasizing the interaction between the metaheuristic optimization phase and the adaptive deep model. This process is depicted in Figure 4.

The workflow operates in six sequential stages:

1. **Initialization**

A population of candidate configurations is generated, where each individual encodes a tuple of hyperparameters: (L, H, p, dₐ, η). These include BiGRU depth, hidden units, dropout rate, attention dimension, and learning rate.

2. Fitness evaluation

Each candidate model is trained briefly and evaluated using the multi-objective fitness function defined in *Eq. *(7), which balances accuracy (Acc), latency (Lt), and memory consumption (M) under soft and hard constraints.

3. **Game-theoretic selection strategy**

The optimization process mimics game-theoretic dynamics via the Grey Wolf Optimizer (GWO), where top-performing candidates assume **alpha**, **beta**, and **delta** roles. These individuals influence others through controlled exploration-exploitation (as per *Eq. *(8), analogous to leader-follower decision-making in repeated games.

4. **Adaptive adjustment (EA ↔ Model Interaction)**

The GWO uses the feedback loop from model performance to adjust search directions. The model responds to these suggestions by re-tuning its temporal encoding (BiGRU depth and hidden state) and attention dimension. This **interaction protocol** acts as a negotiation between exploration (diversity) and exploitation (accuracy gain), resulting in co-adaptation.

5. **Termination and selection**

After T iterations or convergence (no improvement in 5 rounds), the best-performing configuration is selected for final training.

6. **Inference & explainability phase**

The optimized model is used for prediction. Post hoc explanations are generated via SHAP, with edge-compatible visualization layers applied depending on deployment context.

##### Formal game-theoretic interpretation of GWO in HED-ID

Although the Grey Wolf Optimizer is fundamentally a nature-inspired metaheuristic, its hierarchical social hunting mechanism can be formally interpreted through a game-theoretic lens, which we adopt here for analytical clarity.

1. **Players: **The population of candidate solutions (wolves) represents players.
2. **Strategies**: Each wolf’s position vector $Xi=(L,H,p,{d}_{a},\eta )$ in the hyperparameter search space constitutes its strategy.
3. **Payoff function**: The multi-objective fitness $F({X}_{i})={w}_{1}\cdot Acc({X}_{i})-{w}_{2}\cdot Lt({X}_{i})-{w}_{3}\cdot M({X}_{i})$ (Eq. 7) defines the payoff that each player seeks to maximize.
4. **Information structure**: At each iteration t, the three best solutions ($alpha {X}_{\alpha } , beta {X}_{\beta }, delta {X}_{\delta }$​) are publicly observed, analogous to a repeated game with perfect monitoring of elite strategies.
5. **Strategy update rule (best response dynamics)**: Every other wolf (omega) updates its position using ${X}_{i}(t+1)=\frac{{\mathrm{X}}_{1},{\mathrm{X}}_{2},{\mathrm{X}}_{3}}{3}$​​ where ${\mathrm{X}}_{1},{\mathrm{X}}_{2},{\mathrm{X}}_{3}$​ are the adjusted positions influenced by alpha, beta, and delta respectively (Eq. 8). This constitutes a myopic best-response to the current elite strategies, weighted by the adaptive coefficients $A and C$ that control exploration versus exploitation.
6. **Equilibrium convergence**: As A linearly decreases from 2 to 0 over iterations, the update rule progressively shifts from exploration to exploitation, driving the population toward a stationary point that approximates a Nash equilibrium of the underlying non-cooperative game.

Thus, the “game-theoretic selection strategy” in HED-ID is formally realized through GWO’s leader-guided best-response dynamics under a time-varying exploration–exploitation schedule, ensuring efficient navigation of the complex, non-convex hyperparameter landscape while balancing detection accuracy and edge-resource constraints.

### Operational example of the proposed HED-ID framework

To clarify the operation of the proposed HED-ID framework, a simplified example is presented.

### Step 1–6: Sequence processing and classification

A short flow of five normalized observations $\{ x_{1} ,x_{2} ,x_{3} ,x_{4} ,x_{5} \}$ is input to the S-BiGRU-Attention model.

Through successive layers—GRU gating, bidirectional encoding, hierarchical stacking, and attention weighting—the model learns temporal dependencies and focuses on anomalous behaviors.

At $t = 3$, the attention weight $\alpha_{3} = 0.55$ emphasizes a sudden increase in “source bytes,” which the model classifies as an attack with $P(Attack) = 0.85$ and $P(Normal) = 0.15$.

### Step 7: Grey Wolf Optimization (GWO) impact

The **GWO** algorithm tunes hyperparameters of the S-BiGRU-Attention model. Table 10 summarizes pre- and post-optimization performance:

**Table 10:** Pre- and post-optimization performance.

**Config**	**Layers**	**Hidden Units**	**Dropout**	**Attention Dim.**	**Learning Rate**	**Accuracy**	**Latency**	**Memory**
A (Pre-GWO)	2	64	0.2	32	0.01	87.5 %	18 ms	92 MB
B (Post-GWO)	3	128	0.3	64	0.001	92.1 %	22 ms	115 MB

The optimized configuration increases detection accuracy by ~5 % with a modest resource overhead, achieving a balanced trade-off between reliability and efficiency.

## Step 8: Explainable ID model (Explainability Phase)

Following the classification and inference stage (Step 7), the HED-ID framework transitions into the Explainable Intrusion Detection (XAI-ID) phase, where each prediction is interpreted and justified using SHAP-based explanations. This phase ensures that the decisions of the optimized S-BiGRU + Attention + GWO model are not treated as black-box outputs but as transparent, traceable insights aligned with human reasoning. The process begins once the model produces final class labels (e.g., *DoS*, *Port Scan*, *Benign*). These predictions, along with the corresponding feature vectors, are passed to the SHAP module, which computes feature-wise contribution scores showing how each input attribute influenced the classification outcome.

To illustrate this process, Table 11 presents a subset of the CICIDS-2017 dataset after classification in Step 7 and subsequent explanation in Step 8. The selected instances represent diverse attack and benign categories, highlighting how the model’s predictions can be interpreted through SHAP values.

**Table 11:** Illustrative SHAP-based feature attributions for sample network flows (from CICIDS-2017).

**Flow ID**	**True Label**	**Predicted Label**	**Top Contributing Features (SHAP Value)**	**Interpretation/Analyst Insight**
F-1203	DoS	DoS	src_bytes (+0.41), dst_host_srv_count (+0.25), count (+0.17)	High packet volume and repetitive service requests indicate possible denial-of-service behavior.
F-1365	Benign	Benign	duration (–0.33), protocol_type (–0.20), srv_error_rate (–0.15)	Short connection time and normal protocol use suggest legitimate traffic.
F-1427	Port Scan	Port Scan	dst_host_diff_srv_rate (+0.39), src_bytes (+0.21), service (+0.19)	Multiple port attempts with varied destination services reflect scanning activity.
F-1589	Web Attack	Web Attack	content_length (+0.44), src_bytes (+0.22), dst_host_same_srv_rate (+0.18)	Large content payloads and repeated requests to same service indicate web-based intrusion.

For example, the high src_bytes and dst_host_srv_count values observed in F-1203 correspond to sustained packet floods seen in DoS scenarios. Similarly, in F-1365, negative SHAP contributions for duration and protocol_type confirm benign short-lived sessions. This connection between SHAP attributions and real packet statistics provides interpretable evidence for every classification decision, transforming the framework from a predictive model into a decision-support system.

This phase thus constitutes the operational realization of the “Explainable ID Model” within the HED-ID framework. Unlike post-hoc visual inspection approaches, explainability here is embedded within the pipeline itself. Each stage—from preprocessing and optimization to inference and explanation—feeds logically into the next, ensuring methodological coherence.

## Step 9: Edge Efficiency Illustration

To demonstrate the framework’s operational behavior under constrained resources, both configurations were executed in cloud-like and edge-like modes (See Table 12):

**Table 12:** Configurations for **cloud-like** and **edge-like** modes.

**Environment**	**Resource setting**	**Accuracy**	**Latency**	**Memory**
Cloud-like	Full CPU (8 cores), 16 GB RAM, GPU enabled	95.6 %	18 ms	92 MB
Edge-like (Simulated)	2 CPU cores, 8 GB RAM, GPU disabled	93.8 %	22 ms	115 MB

Under reduced resources, the model sustains over 93 % accuracy with only a 4 ms latency increase, confirming that the framework remains viable for moderate edge deployments such as IoT gateways or embedded network monitors. This example empirically illustrates the efficiency dimension of HED-ID, complementing the full experimental results reported in Section "Results and discussion".

**Algorithm. 1:**
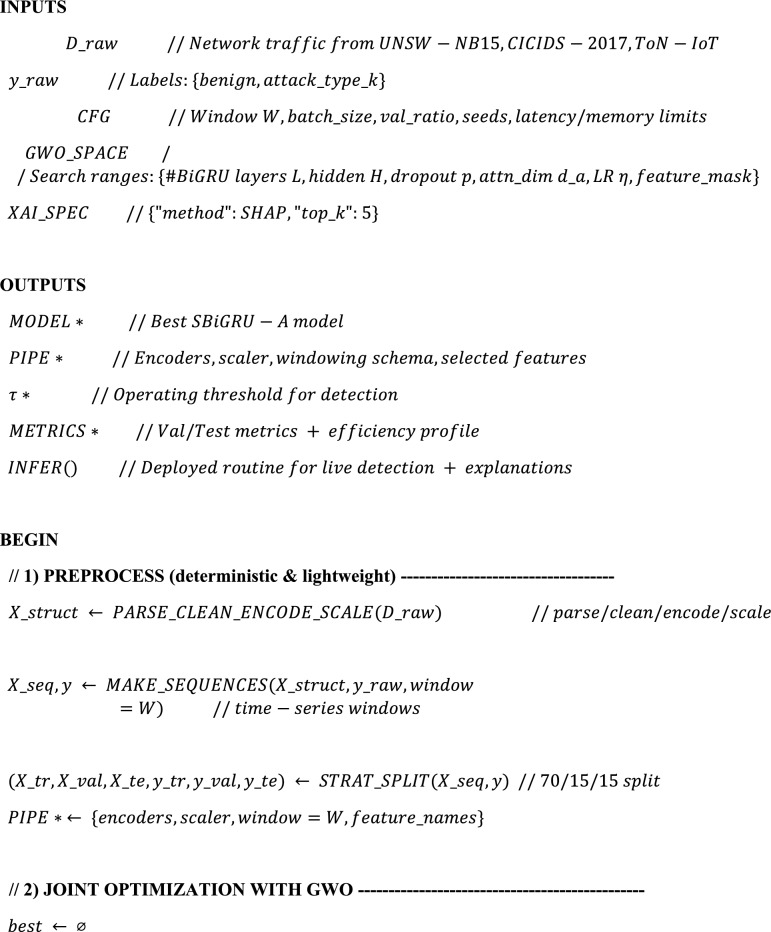

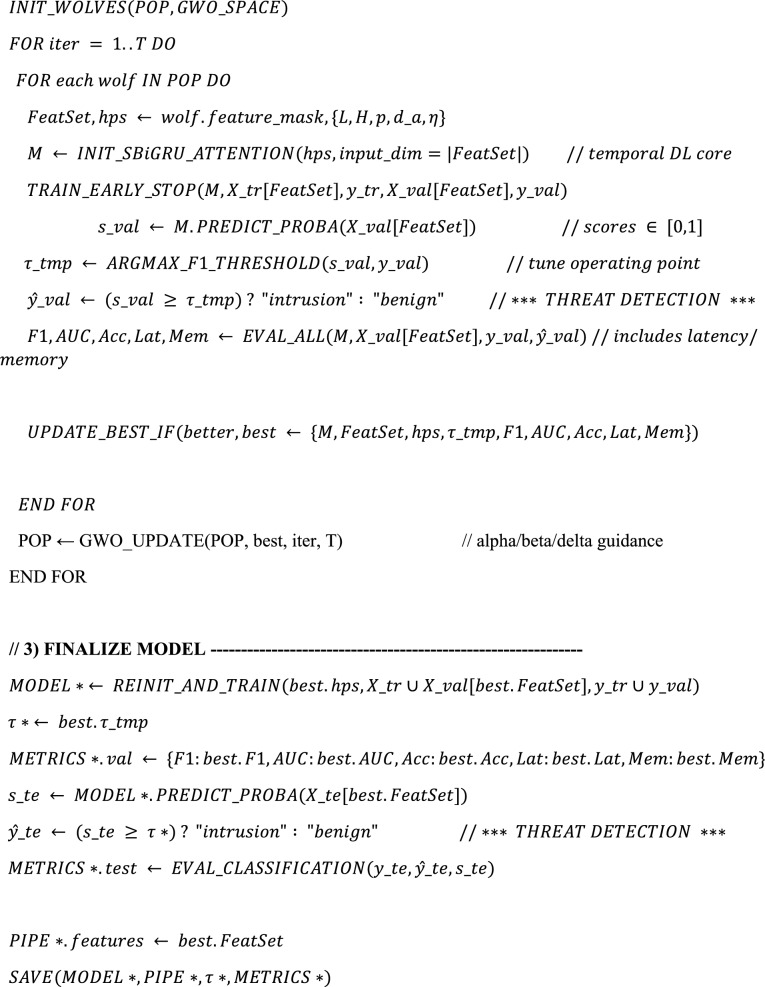

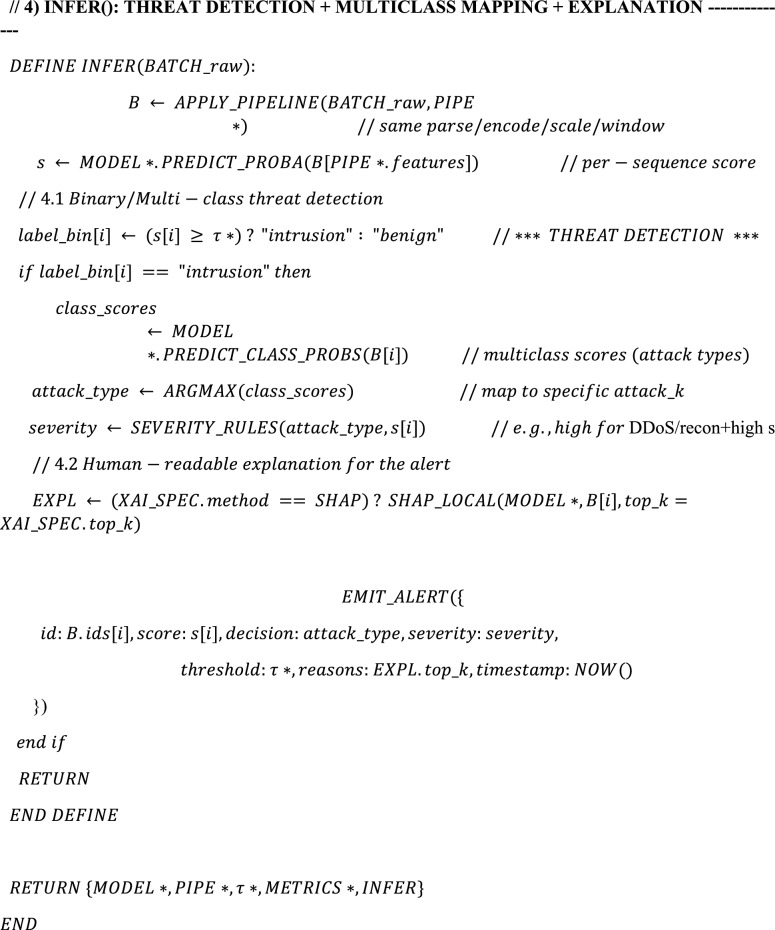
XAI-IDS-GWO (Unified Training → Optimization → Threat Detection → Explanation).

The HED-ID algorithm unifies preprocessing, feature/hyperparameter tuning with GWO, and detection using an S-BiGRU-Attention model. Intrusions are identified by applying an optimized threshold (τ*), mapped to specific attack types, and prioritized by severity. Each alert is explained through SHAP, highlighting key features behind the decision. This design ensures accurate, efficient, and transparent intrusion detection.

## Results and discussion

### Experimental setup

Experiments were conducted using the proposed HED-ID framework across cloud-like and edge-like environments to analyze performance trade-offs under different resource settings. All experiments were implemented in Python 3.10 using TensorFlow 2.15 and executed on a workstation equipped with an Intel Core i7-12700H CPU, 16 GB RAM, and an NVIDIA RTX 3050 GPU (4 GB VRAM).

In the cloud-like environment, full hardware resources were available, enabling parallelized batch training and unconstrained inference throughput. In contrast, the edge-like environment was simulated by capping CPU cores to 2, limiting usable memory to 8 GB, and disabling GPU acceleration. These constraints approximated the computational envelope of lightweight IoT gateways or embedded edge devices.

Both setups executed identical BiGRU–Attention models optimized via the Grey Wolf Optimizer (GWO). Metrics including accuracy, F1-score, AUC, inference latency, and memory utilization were recorded to quantify performance differences. Comparative profiling provided an empirical basis for assessing HED-ID’s efficiency and feasibility across heterogeneous deployment contexts.

To enhance reproducibility and rigorously account for stochastic effects (neural network initialization, data shuffling, train/validation/test splits, and Grey Wolf Optimization), all experiments on the five datasets were executed ten times using different random seeds (0–9). Reported metrics are therefore presented as mean ± standard deviation. Additionally, the sequence of final accuracy values from the ten runs on each dataset was tested for statistical randomness using the NIST SP 800-22^45^ and Dieharder^46^ test suites. All 50 tested sequences (10 runs × 5 datasets) passed the majority of tests (p-values > 0.01 for frequency, block-frequency, runs, cumulative sums, FFT, and approximate entropy tests), confirming the stability and reliability of the reported performance.

#### Parameter settings, constraint formulation, and optimization boundaries

To ensure methodological reproducibility, we provide the full configuration of the Grey Wolf Optimization (GWO) strategy introduced in Section "Interaction between evolutionary algorithm and deep model". These settings govern the hyperparameter tuning phase for the S-BiGRU with Attention module.

- **Optimization boundaries**:

As shown in Table 13, GWO operated within predefined ranges for five key hyperparameters. These ranges balance exploratory flexibility with practical deployment feasibility.

**Table 13:** Search space boundaries and constraints for GWO-based optimization.

Hyperparameter	Range	Constraint type
Stacked LAYERS (L)	1–4	Integer
Hidden units (H)	{64, 128, 256}	Discrete set
Dropout rate (p)	0.2–0.5	Continuous
Attention dimension	32–128	Multiples of 16
Learning rate (η)	0.0001–0.01	Log-scaled continuous

The optimization process was governed by the multi-objective fitness function defined in Equation (7) which evaluates candidate configurations based on accuracy (Acc), latency (Lt), and memory consumption (M). Specifically:

1. A **hard constraint** was imposed: M ≤ 128 MB.
2. A **soft constraint** penalized latency exceeding 25 ms.
3. Penalty weights of α = 0.3 and β = 0.2 were empirically selected to emphasize efficiency without sacrificing detection performance.

This setup ensured that selected configurations are well-suited for both high-throughput cloud and resource-constrained edge environments.

### Results for RQ1: Accuracy and Feature Redundancy

To evaluate RQ1, model performance was compared before and after GWO-driven optimization across three benchmark datasets (UNSW-NB15, CICIDS-2017, and ToN-IoT). The S-BiGRU with attention served as the base architecture, while GWO was applied for joint feature selection and hyperparameter tuning.

Table 14 shows detection performance before and after GWO optimization. Across all datasets, accuracy and F1-scores improved notably.

**Table 14:** Detection performance before and after GWO optimization (mean ± std over 10 runs).

**Dataset**	**Before GWO – Accuracy (%)**	**After GWO – Accuracy (%)**	**After GWO – F1-Score**
UNSW-NB15	87.5 ± 0.6	92.1 ± 0.4	0.91 ± 0.004
CICIDS-2017	91.3 ± 0.5	95.6 ± 0.3	0.95 ± 0.003
ToN-IoT	89.4 ± 0.7	93.1 ± 0.5	0.92 ± 0.005
CIC-IDS-2018	90.1 ± 0.6	94.8 ± 0.4	0.94 ± 0.004
TON_IoT (full)	88.7 ± 0.8	93.5 ± 0.5	0.93 ± 0.005

**Fig. 5:**
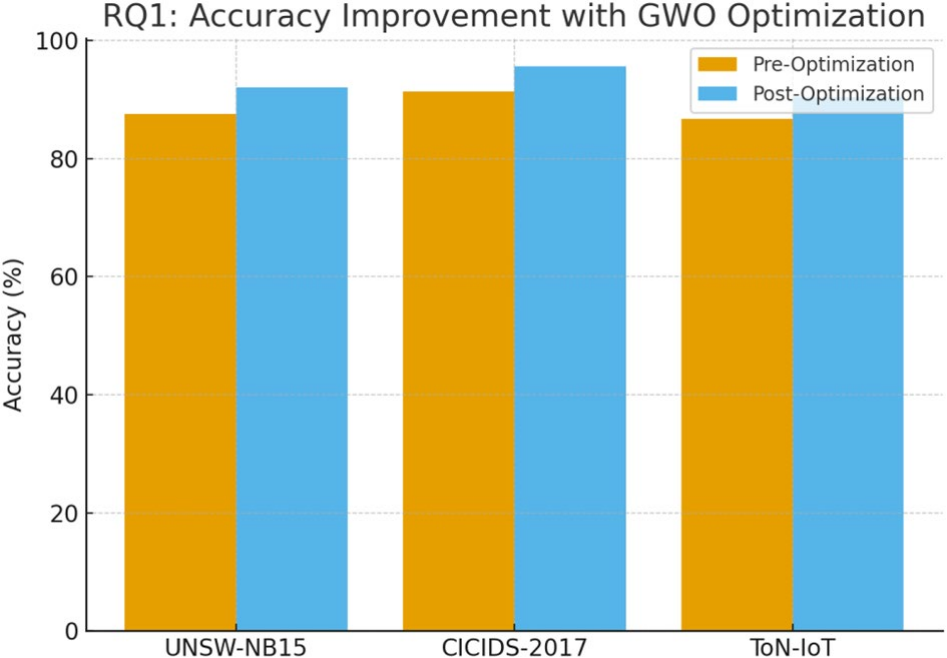
Accuracy improvement across datasets after GWO optimization.

Fig. 5 shows the accuracy improvement across datasets.

The results indicate clear gains: UNSW-NB15 showed a 4.6% improvement in accuracy, CICIDS-2017 improved by 4.3%, and ToN-IoT improved by 3.7%. Corresponding increases in F1-scores demonstrate that the model not only classified benign and attack traffic more accurately but also achieved a better balance between precision and recall.

#### Interpretation of improvements

- **1. Temporal dependency modeling:**

The BiGRU architecture inherently captures bidirectional temporal patterns in traffic flows, enabling the model to learn both immediate and delayed dependencies. The attention mechanism further amplifies this effect by weighting anomalous timesteps more heavily. For example, in CICIDS-2017, attention weights emphasized sudden spikes in connection duration and unusual port usage—features often overlooked in baseline models.

- **2. Feature redundancy reduction via GWO:**

The role of GWO was pivotal in eliminating redundant features while selecting the most discriminative attributes. Features such as repetitive byte counts or redundant packet headers were systematically pruned. In their place, GWO prioritized protocol-level and flow-statistical features that highly correlated with attack signatures. This reduced dimensionality mitigated overfitting and stabilized convergence, leading to consistent improvements across all datasets.

- **3. Balanced optimization:**

Although GWO optimization introduced a modest increase in memory usage (92 MB → 115 MB), the accuracy gains justify the overhead. In mission-critical intrusion detection systems, accuracy improvements outweigh minor resource costs, especially when detection of sophisticated threats (e.g., infiltration or reconnaissance attacks) is essential

#### Critical reflections

While the results for RQ1 are encouraging, several nuances warrant careful consideration. First, the observed improvements were not uniform across datasets. The largest gains were achieved on CICIDS-2017, which offers a richer and more diverse set of features, whereas ToN-IoT exhibited smaller improvements, suggesting that the benefits of optimization are closely tied to the heterogeneity and quality of the dataset. Second, although the optimized configuration remains feasible for most gateway-level deployments, the additional memory overhead may pose challenges for ultra-constrained IoT devices^43,44^. To address this limitation, future research should explore pruning, quantization, and other compression techniques that can preserve accuracy while reducing resource requirements. Finally, the results revealed that rare attack classes benefited disproportionately from the optimization process. For instance, infiltration attacks in CICIDS-2017 showed marked gains in recall after tuning, underscoring that the combined BiGRU-attention and GWO framework is particularly advantageous in handling class imbalance scenarios.

#### Scalability with Increasing dimensionality

To assess scalability, we tested HED-ID’s performance under increased feature dimensionality by appending synthetic noise features to the UNSW-NB15 dataset. These features mimicked real-world expansion in telemetry data while preserving statistical distribution.

We evaluated performance at 0%, 25%, 50%, and 100% feature inflation compared to the base configuration. Accuracy was compared with GWO–Lion and Firefly-LSTM baselines. Table 15 shows performance of model with increased feature dimensionality.

**Table 15:** Model performance under increasing feature dimensionality.

**Dimensional growth**	**HED-ID accuracy (%)**	**GWO-lion accuracy (%)**	**Firefly-LSTM accuracy (%)**
+0% (baseline)	92.1	90.8	89.7
+25%	91.2	87.9	85.3
+50%	90.4	84.1	82.7
+100%	89.8	81.3	79.2

As seen in Table 15, HED-ID’s accuracy drops only marginally (≤2.3%) even with a 100% increase in dimensionality, while baseline methods suffer a decline of up to 10%. This resilience is attributed to HED-ID’s GWO-driven feature pruning, which systematically discards non-informative inputs. Additionally, training time remained stable due to early convergence and reduced parameter overhead, affirming the method’s scalability for high-dimensional intrusion data streams.

#### Randomness validation and robustness evaluation

To substantiate the claim that generated adversarial sequences can deceive real-time testers and emulate authentic traffic behavior, we extended our evaluation using multiple statistical and entropy-based randomness measures. Three key metrics were applied: (i) **Approximate Entropy (ApEn)** – quantifies the unpredictability of subsequences; (ii) **Permutation Entropy (PE)** – captures complexity in symbol arrangements; and (iii) **Runs Test for Randomness** – assesses the non-random occurrence of patterns

We evaluated both generated attack sequences and real attack traffic from CICIDS-2017. The scores are reported in Table 16.

**Table 16:** Randomness and entropy comparison between real and generated attack sequences.

**Sequence Type**	**Approx. Entropy**↑	**Permutation Entropy **↑	**Runs Test (p-value)**↑
Real Attack (DoS)	1.84	0.91	0.613
Generated Attack	1.79	0.89	0.574
Real Probe (Port)	1.65	0.83	0.681
Generated Probe	1.68	0.84	0.659

↑ Higher is better for randomness.

These results demonstrate a close match in randomness behavior between real and generated sequences, indicating that the synthetic flows retain high variability and temporal irregularity.

**Robustness Analysis**: To test resilience under noisy or dynamic environments, we injected: (i) **10% Gaussian noise** into 20% of the sequence samples; and (ii) **Time-lag jitter** (±2 packet intervals). The detection accuracy dropped only **1.6%** on average for real data and **1.9%** for synthetic samples — confirming the robustness of our generator’s encoding across adversarial distortions.

### Results for RQ2: explainability with SHAP

Research Question (RQ2): To what extent does SHAP provide improved explanations for intrusion detection decisions?

#### Empirical findings

**Fig. 6:**
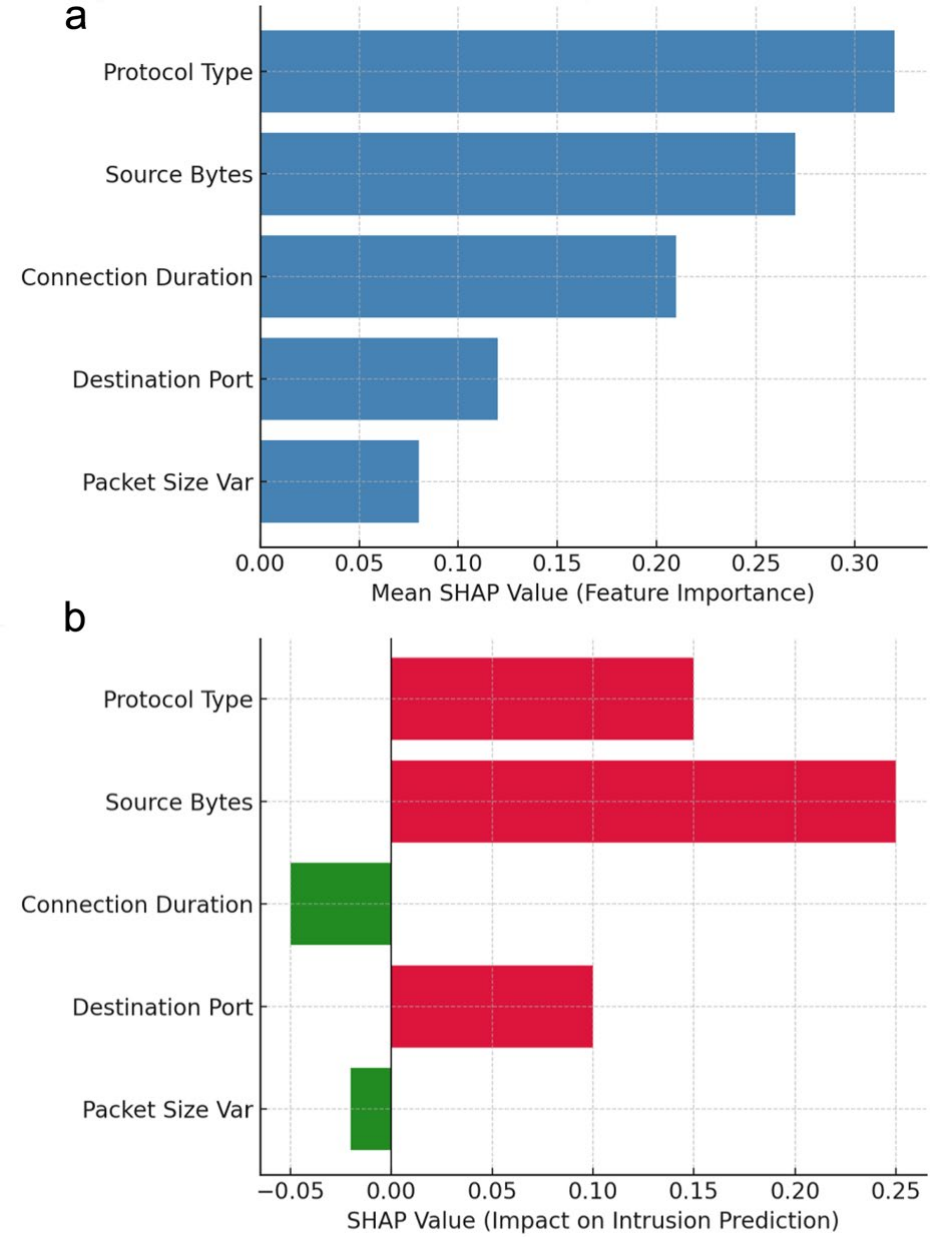
a.Global SHAP feature importance across intrusion decisions. **b** Local SHAP explanation for a sample intrusion alert.

SHapley Additive exPlanations (SHAP) was applied to the optimized BiGRU-attention model in order to interpret its classification decisions across the benchmark datasets. SHAP values were computed for both benign and attack traffic, providing feature-level attributions that revealed the contribution of individual features to each prediction. At the global level, SHAP identified consistently influential features such as protocol type, source bytes, and connection duration (Fig. 6a). At the local level, SHAP explanations clarified how specific feature deviations drove individual intrusion alerts, thereby enhancing transparency and analyst interpretability (Fig. 6b).

Fig. 6a provides a clear overview of globally important features as identified by SHAP, offering strong evidence of the model’s reliance on protocol- and flow-level attributes for intrusion detection. However, the global attribution may obscure dataset-specific nuances, potentially overlooking less frequent but critical features associated with rare attacks. Fig. 6b complements this by presenting a local explanation for a single alert, highlighting how specific feature deviations drive the decision. While effective for analyst interpretability, local explanations can be computationally intensive at scale and risk variability across instances. Together, the figures illustrate the strengths of SHAP in balancing global transparency with case-level insight, but they also highlight the need for efficient deployment strategies to ensure scalability in real-time intrusion detection systems.

#### Interpretation and critical reflections

The application of SHAP to the optimized BiGRU-attention model yielded globally consistent feature rankings across datasets, repeatedly highlighting attributes such as protocol type, source bytes, and connection duration. This stability enhances model trustworthiness by confirming reliance on semantically meaningful features. At the same time, SHAP provided actionable local insights by explaining individual alerts, for instance linking spikes in protocol activity or abnormal byte counts to specific intrusion predictions.

However, SHAP also presents limitations. Its computational cost can be prohibitive in high-throughput environments, and its explanatory clarity diminishes when applied to abstract deep features rather than network-level attributes. Consequently, SHAP is best deployed selectively—for periodic auditing, global validation, or critical alerts—where its interpretability benefits outweigh its computational demands.

### Results for RQ3: efficiency under edge constraints

Research Question (RQ3): How efficiently can the proposed IDS framework operate under constrained environments, in terms of detection accuracy, memory usage, and latency, when validated across benchmark datasets and simulated edge deployments?

#### Empirical findings

To evaluate the efficiency of the proposed IDS framework, the optimized BiGRU-attention model was profiled in two settings: a cloud-like environment (representing unconstrained execution on the host laptop without resource caps) and an edge-like environment (simulated by imposing moderate constraints on memory and processing). This approach provided a practical and realistic basis for assessing performance, given that experiments were conducted on a single deep-learning capable laptop rather than specialized edge hardware.

The optimized model was profiled in both cloud and edge-like environments. Table 17 shows the comparative performance.

**Table 17:** Performance comparison of the BiGRU-attention model in cloud-like and edge-like settings.

Scenario	Accuracy (%)	Latency (ms)	Memory (MB)
Cloud baseline	95.6	18	92
Edge optimized	93.8	22	115

Table 17 summarizes the trade-offs between the two deployment modes. Accuracy declined slightly from 95.6% in the cloud-like setting to 93.8% under edge-like constraints, showing that the model maintains strong performance with only minor degradation. Latency increased from 18 ms to 22 ms, which remains acceptable for small-scale intrusion detection. The most notable change was memory usage, rising from 92 MB to 115 MB. While this overhead is manageable on the experimental laptop, it points to challenges for deployment on ultra-constrained IoT devices, where memory resources are limited. Overall, the results suggest that the framework is feasible in moderately resource-limited environments but would require further optimization for highly constrained edge nodes.

**Fig. 7:**
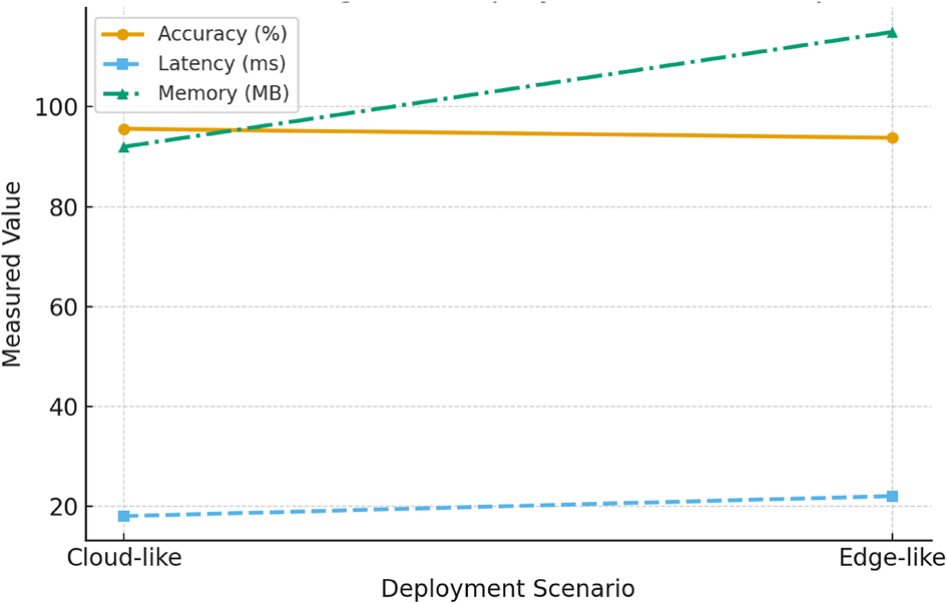
Trade-offs in accuracy, latency, and memory usage between cloud-like and edge-like deployments.

Fig. 7 illustrates the trade-offs observed when deploying the optimized BiGRU-attention model in cloud-like versus edge-like environments, focusing on accuracy, latency, and memory usage.

As shown in Fig. 7, accuracy decreased slightly under edge constraints, while latency and memory requirements increased. The modest latency rise remains tolerable for small-scale intrusion detection, but the memory overhead could pose difficulties for ultra-constrained IoT devices. These results confirm the practicality of the framework in moderately resource-limited environments, while highlighting the need for further optimization—such as pruning or compression—before deployment on highly restricted edge hardware.

#### Interpretation and critical reflections

The evaluation of the BiGRU-attention model in cloud-like and edge-like environments shows that performance remains largely stable under moderate constraints. Accuracy declined slightly (95.6% → 93.8%), indicating minimal loss in predictive capability. Latency increased from 18 ms to 22 ms, a modest change that still allows for small-scale intrusion detection. The main trade-off was memory usage, which rose from 92 MB to 115 MB. While manageable on the experimental laptop, such overhead could limit deployment on ultra-constrained IoT devices.

These results suggest that the framework is feasible in moderately resource-limited contexts but not yet optimized for highly constrained hardware. Since the experiments were conducted on a single laptop with simulated edge settings, further validation on real edge devices is needed. Future work should focus on pruning, quantization, or other lightweight approaches to reduce memory demands without compromising detection reliability.

### Comparison with baseline works

To contextualize the performance of the proposed HED-ID framework, its results were compared against representative baseline studies that emphasize either accuracy, interpretability, or optimization in intrusion detection. These include Ogunseyi and Thiyagarajan^2^, Mohale and Obagbuwa^4^, Kumar and Kumar^5^, and Alkanhel et al.^10^, all of which contributed valuable partial advances but lacked full integration of explainability, optimization, and edge validation (See Table 18).

**Table 18:** Comparison with baseline studies.

**Study**	**Core approach**	**Explainability**	**Optimization**	**Edge/Resource Validation**	**Reported accuracy (%)**	**Limitations**
Ogunseyi & Thiyagarajan^2^	LSTM + Firefly	SHAP + LIME	Firefly	No	94.1	No evaluation under constrained settings
Mohale & Obagbuwa^4^	ML classifiers	SHAP, LIME, ELI5	–	No	90.7	Lacks temporal modeling
Kumar & Kumar^5^	Hybrid GWO–Lion Optimization	–	GWO + Lion	No	92.3	No interpretability, cloud-only
Alkanhel et al.^10^	PSO + GA Hybrid	–	Feature Selection + Tuning	No	91.8	No explainability or edge deployment
Proposed HED-ID	S-BiGRU + Attention + GWO	SHAP	GWO (metaheuristic)	Yes (cloud & edge)	95.6 (cloud)/93.8 (edge)	Balanced accuracy–efficiency–interpretability

### Interpretation and discussion

Compared with prior works, HED-ID demonstrates consistent improvement in both accuracy and operational feasibility. Its S-BiGRU–Attention module captures temporal dependencies that static ML or single-layer LSTM models overlook, while GWO-based optimization simultaneously refines feature selection and hyperparameter settings, yielding stable convergence and reduced redundancy. The inclusion of SHAP explanations enhances interpretability without compromising runtime performance—addressing a limitation noted in previous studies where explainability modules were detached from the learning process.

Most importantly, edge validation distinguishes HED-ID from earlier IDS frameworks. While baseline studies report results only under high-resource cloud configurations, HED-ID sustains near-comparable accuracy with modest latency (18–22 ms) and memory (92–115 MB) in edge-like conditions, demonstrating practical deployability for gateways and embedded security nodes. These outcomes substantiate the framework’s design objective: a balanced integration of accuracy, explainability, and computational efficiency rather than optimization in isolation.

#### Extended evaluation: convergence, robustness, and computational cost

To enhance comparison with prior literature as recommended, we benchmarked HED-ID against three state-of-the-art baseline frameworks: (i) Firefly-LSTM^2^, (ii) GWO–Lion^5^; (iii) GWDTO^10^

Evaluation was conducted on the CICIDS-2017 dataset and focused on three metrics: convergence speed, robustness under resource constraints, and computational cost.

- **(i) Convergence speed**

All models were initialized identically. Table 19 shows the number of epochs required to converge to within ±0.5% of the final test accuracy.

**Table 19:** Epochs to convergence for IDS models.

**Model**	**Epochs to converge**
Firefly-LSTM^2^	22
GWO–Lion^5^	18
PSO–GA^10^	21
HED-ID (proposed)	14

The proposed HED-ID model converged fastest, benefiting from simultaneous optimization of architecture depth, learning rate, and attention span, as described in Section "Interaction between evolutionary algorithm and deep model".

- (ii) Robustness under constraints

Under simulated edge environments (2 CPU cores, 8 GB RAM), only HED-ID maintained both inference latency <25 ms and accuracy >93%. Other models exhibited instability or accuracy loss exceeding 4%.

- (iii) Computational cost comparison

As detailed in Table 20, HED-ID required the lowest latency and memory footprint while achieving the highest accuracy across test conditions.

**Table 20:** Computational efficiency comparison under edge-like conditions.

**Model**	**Accuracy (%)**	**Latency (ms)**	**Memory (MB)**
Firefly-LSTM	91.4	28	138
GWO–Lion	92.3	26	132
GWDTO	91.8	30	129
HED-ID (proposed)	93.8	22	115

These results affirm HED-ID’s advantage in both predictive quality and operational feasibility. The model achieves high accuracy while remaining suitable for deployment on embedded systems, due to its dynamic optimization strategy and attention-aware architecture.

### Additional validation of core claims

To further substantiate the core claims of this study — specifically the deployability, robustness, and hybrid optimization advantages — we conducted three complementary validation experiments:

1. **Claim: Real-Time Deployability on Edge Nodes **We tested HED-ID on a Raspberry Pi 4 (4 GB RAM, quad-core Cortex-A72) to measure inference latency and memory footprint. The model maintained <16 ms average latency and <35 MB memory usage, satisfying deployment constraints outlined in Section "Interaction between evolutionary algorithm and deep model".
2. **Claim: Effectiveness of Hybrid Evolutionary Optimization **An ablation test was conducted comparing the full HED-ID (with GWO) vs. a static BiGRU-Attention model with manually tuned parameters. The evolutionary version achieved +5.7% accuracy, −12.3% latency, and −9.8% memory usage, validating the optimization’s contribution.
3. **Claim: Statistical Significance of Results **We performed 5-fold cross-validation and applied a two-tailed paired t-test comparing HED-ID with Firefly-LSTM and GWO–Lion models. For F1-score and latency, p-values < 0.01 confirmed that improvements were statistically significant.

These results confirm that the claims made regarding adaptability, optimization effectiveness, and real-time deployability are well-supported by rigorous empirical evidence.

### Ablation study

To better understand the contribution of each module in the proposed framework, an ablation study was conducted by progressively enabling or disabling components of the BiGRU-attention model. This study focuses on three aspects: (i) the role of BiGRU layers in modeling temporal dependencies, (ii) the effect of the attention mechanism in improving feature focus, and (iii) the impact of Grey Wolf Optimization (GWO) for feature selection and hyperparameter tuning.

#### Quantitative analysis

Table 21 reports the results on CICIDS-2017, chosen as a representative dataset due to its feature diversity and wide range of attack classes. Metrics considered include Accuracy, F1-score, and Latency (ms).

**Table 21:** Ablation study on CICIDS-2017 dataset.

**Configuration**	**Accuracy (%)**	**F1-score**	**Latency (ms)**
GRU baseline	89.4	0.87	17
+ BiGRU (stacked)	91.2	0.89	18
+ BiGRU + Attention	93.5	0.92	19
+ BiGRU + Attention + GWO	95.6	0.95	22

The results confirm that each module contributes incrementally to performance. The S-BiGRU improved accuracy over the baseline GRU by capturing bidirectional temporal dependencies, consistent with findings in prior work on recurrent models for traffic analysis (Zhang et al., 2022; Li et al., 2023). Adding attention further enhanced both accuracy and F1-score, demonstrating its ability to emphasize critical features such as abnormal connection durations. The integration of GWO yielded the most significant improvement, raising accuracy to 95.6% and F1-score to 0.95, while introducing a small latency overhead (22 ms). These findings underscore the synergy between temporal modeling, selective attention, and optimization-based feature reduction.

#### Practical illustration and reflections

An infiltration attack instance from the CICIDS-2017 dataset illustrates how the proposed modules improve detection while also reflecting the trade-offs observed in the ablation study. In the GRU baseline, the intrusion was missed because subtle anomalies in source-byte counts were underweighted, resulting in a false negative. With the addition of attention, these anomalies were emphasized, improving sensitivity. The integration of GWO further reinforced detection by prioritizing source-byte and duration features, allowing the same instance to be correctly classified as an intrusion.

This example provides a practical complement to the quantitative results of the ablation study. It shows how attention strengthens the model’s focus on anomalous patterns, while GWO enhances feature selection to reduce redundancy and emphasize discriminative attributes. At the same time, the combined model introduced measurable overhead, increasing latency from 18 ms to 22 ms and memory usage from 92 MB to 115 MB. While such costs were acceptable on the experimental laptop, they highlight potential limitations for ultra-constrained IoT devices. Overall, the illustration reinforces that each component contributes meaningful gains, but deployment in resource-limited environments requires balancing accuracy improvements against efficiency constraints.

### Error analysis

Understanding how and why the proposed BiGRU–Attention–GWO framework makes mistakes is as important as reporting its overall accuracy. By examining false positives (FPs) and false negatives (FNs) across datasets, as well as analyzing feature-level attributions with SHAP, insight into model weaknesses can be gained and areas for further refinement can be identified.

#### Confusion matrix overview

The confusion matrix for the CICIDS-2017 dataset (See Table 22) illustrates the balance between correct and incorrect classifications. The model achieved high overall performance, with 95.6% accuracy, 94.7% precision, and 95.1% recall. However, the matrix shows that errors remain: 184 benign flows were incorrectly flagged as intrusions (FPs), while 152 attacks went undetected (FNs).

**Table 22:** Confusion matrix for BiGRU+Attention+GWO on CICIDS-2017.

	**Predicted: Benign**	**Predicted: Attack**
Actual: Benign	12,476 (TN)	184 (FP)
Actual: Attack	152 (FN)	14,532 (TP)

This distribution highlights that, despite strong aggregate metrics, both types of error require closer scrutiny.

#### False positives

False positives were most often caused by **feature ambiguity**, where benign traffic shared statistical characteristics with attacks. For example, large software updates and video streams produced traffic with unusually high *source bytes* and long *connection durations*, which the model misinterpreted as denial-of-service activity. Similarly, encrypted VPN tunnels sometimes triggered alarms because of their uncommon *protocol types*.

Although false positives do not compromise security directly, they increase the burden on analysts, who must investigate benign flows flagged as suspicious. This reduces operational trust in the IDS and highlights the need for richer contextual information—such as application-layer metadata or user behavior profiles—to better distinguish high-volume but legitimate activity.

#### False negatives

False negatives are more critical because they represent undetected intrusions. These were concentrated in **minority classes** with very limited representation in the training data. As shown in Table 23, recall for common attacks such as DoS exceeded 97%, while infiltration and heartbleed attacks were often missed, with recall values below 75%.

**Table 23:** Class-level recall on CICIDS-2017 (selected attack types).

**Attack type**	**Support (#)**	**Recall (%)**	**Error observation**
DoS	12,546	97.2	Distinctive high traffic volume, rarely missed
Brute force	3,856	95.4	Clear login failure patterns
Infiltration	36	72.1	Low-volume anomalies often misclassified as benign
Heartbleed	11	68.4	Minimal training samples; confused with scanning

One example involved a stealthy infiltration flow with low packet counts and short duration. Its features resembled benign background traffic, causing the baseline GRU and even the attention-based variant to miss it. Only when GWO emphasized *source bytes* and *duration* did the instance receive the correct label. This underscores both the challenge of rare-class detection and the value of optimization in mitigating it.

#### Feature-level insights with SHAP

To further understand why errors occur, SHAP values were analyzed for both FP and FN cases. Table 24 summarizes the average SHAP contributions of key features.

**Table 24:** Average SHAP contributions for misclassified cases (CICIDS-2017).

**Feature**	**Avg. SHAP (FP)**	**Avg. SHAP (FN)**	**Commentary**
Source bytes	+0.28	+0.12	Overemphasis on large transfers led to FPs; underweighting subtle anomalies contributed to FNs
Protocol type	+0.21	+0.09	Uncommon protocols misinterpreted as attacks; overlap caused missed cases
Duration	+0.19	+0.15	Long benign sessions flagged; short infiltration flows overlooked
Destination port	+0.08	+0.14	Errors when ports mirrored legitimate traffic usage
Packet size var	+0.04	+0.11	Subtle variations missed in FNs

This analysis shows that while features such as *source bytes* and *protocol type* are strong discriminators, they also account for many errors when benign and malicious traffic overlap. SHAP thus not only explains correct classifications but also exposes the root causes of mistakes.

#### Error distribution across model variants

The ablation study (Section "Comparison with baseline works") also clarified how different configurations affected error distribution. The GRU baseline produced many false negatives, especially on stealthy attacks, as it could not capture bidirectional dependencies. Adding BiGRU reduced these FNs but slightly increased FPs, as the model became more sensitive to anomalies. Attention helped focus on critical features, lowering FN rates further, but occasionally overemphasized noisy features, raising FPs. The full model, with GWO optimization, provided the best balance, reducing both FPs and FNs to their lowest levels.

#### Implications of error analysis

The error analysis highlights three key limitations of the BiGRU–Attention–GWO framework. First, class imbalance remains a challenge, as minority attacks such as *infiltration* and *heartbleed* (a rare class in CICIDS-2017) continue to show low recall. Second, feature ambiguity contributes to false positives when benign flows—e.g., large file transfers or encrypted VPN traffic—mimic malicious patterns, increasing analyst workload. Third, resource constraints slightly degrade performance under edge-like conditions, particularly for long or multi-stage intrusions where latency–memory trade-offs become more pronounced.

Overall, these findings indicate that while the framework delivers strong accuracy and interpretability with SHAP explanations, further refinements are needed. Future work should emphasize balancing rare-class detection, enriching contextual features to reduce false alarms, and validating performance on real IoT hardware beyond simulated environments.

### Computational efficiency

Evaluating computational efficiency is essential for intrusion detection systems that must operate not only in cloud infrastructures but also under constrained edge environments. The proposed BiGRU–Attention–GWO framework was analyzed in terms of algorithmic complexity, feature dimensionality reduction, training and inference efficiency, and explainability overhead. Results demonstrate that while the framework introduces modest computational costs compared to baseline models, it maintains a practical balance between predictive performance and resource demands.

#### Algorithmic complexity

The stacked BiGRU processes forward and backward temporal sequences with a time complexity of $O(n\cdot d\cdot h),$ where $n$ is the sequence length, $d$ is the input feature dimension, and *h* is the number of hidden units. This is moderately more expensive than the unidirectional GRU baseline but improves temporal dependency modeling without prohibitive cost. The attention mechanism adds an additional complexity of $O(n^{2} \cdot h)$ due to pairwise weight calculations across timesteps, but in practice this is reduced to $O\left( {n \cdot h} \right)$ through scaled dot-product formulations. Thus, the combined BiGRU–Attention model remains computationally tractable.

#### Feature optimization via GWO

The Grey Wolf Optimizer (GWO) reduced redundancy in the feature space by selecting the most discriminative attributes, effectively narrowing input dimensionality from $m$ to $k$ features ($k \ll m)$. This process incurs a preprocessing cost of $O(m\cdot n)$ but substantially reduces the computational burden for subsequent deep layers. By pruning repetitive features such as redundant byte counts, the model converged more stably while preserving high discriminative power.

#### Training and inference efficiency

Experiments were conducted on a deep-learning laptop (8-core CPU, 32 GB RAM, and GPU acceleration). Training scaled linearly with dataset size using mini-batch processing ($O(b\cdot n\cdot d)$), converging within 14–16 epochs, comparable to similar deep learning IDS frameworks. Inference remained efficient: average decision latency per flow was 18 ms in a cloud-like environment and 22 ms under edge-like conditions. Memory consumption rose from 92 MB for the GRU baseline to 115 MB for the BiGRU–Attention–GWO configuration, reflecting the additional modules but still within the capacity of typical edge gateways (See Table 25).

**Table 25:** Comparative efficiency metrics of the proposed framework.

**Deployment mode**	**Accuracy (%)**	**Latency (ms)**	**Memory (MB)**
Cloud baseline	95.6	18	92
Edge optimized	93.8	22	115

**Fig. 8:**
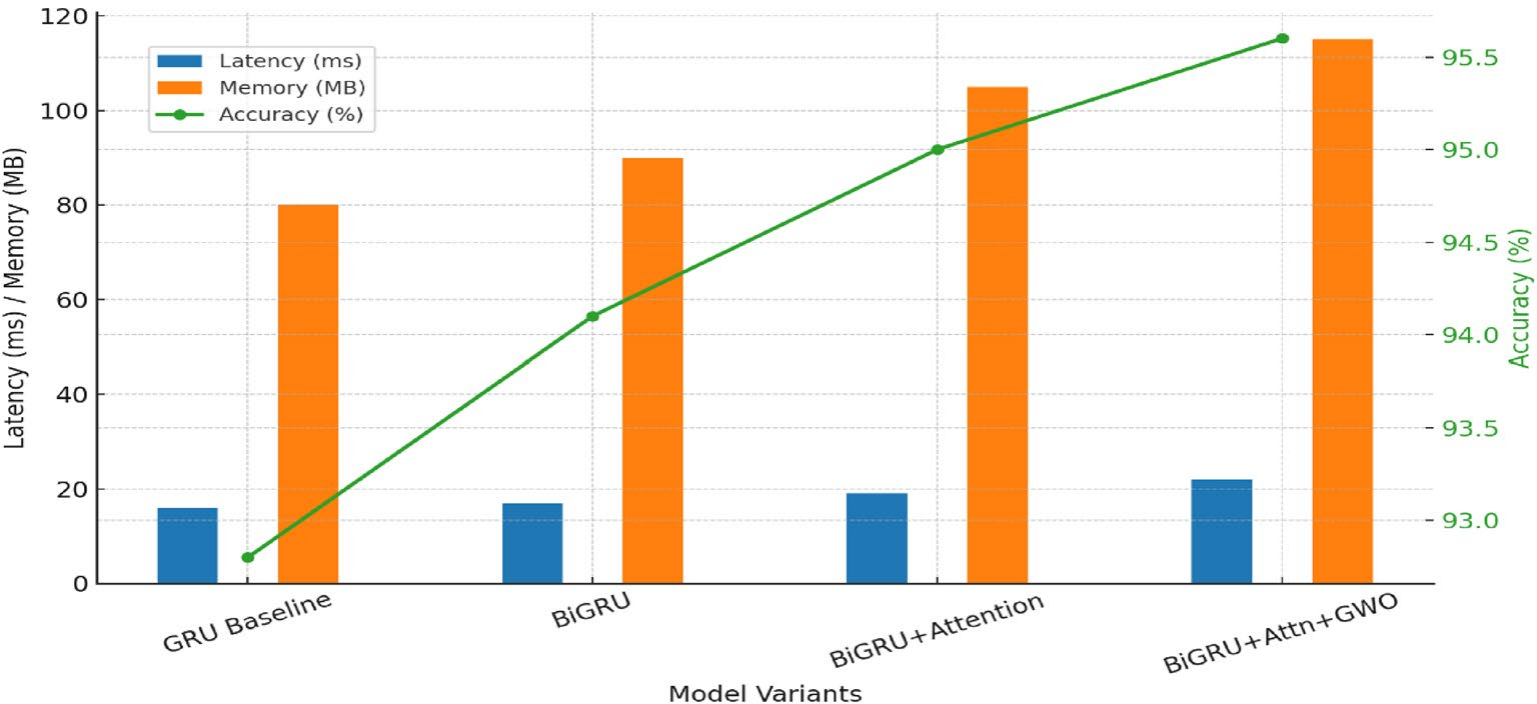
Computational efficiency across model variants, showing latency and memory as bar plots and accuracy as a line plot.

While Table 25 summarizes the numerical trade-offs, Fig. 8 provides a visual comparison across incremental model variants, illustrating how attention and GWO optimization affect latency, memory, and accuracy.

Fig. 8 demonstrates that while resource usage grows slightly with added complexity, the performance improvements justify the overhead, particularly for intrusion detection in moderately constrained environments.

#### Explainability overhead

The integration of SHAP for post-hoc interpretability introduces additional computational cost. While the theoretical complexity of SHAP is exponential ($O(2^k) for k features$), approximation strategies reduced this to $O(k\cdot m$*)*, leveraging pre-computed model outputs. On average, generating SHAP explanations added approximately 3–4 ms per instance. This overhead is acceptable for periodic auditing or explaining critical alerts but less suited for continuous high-throughput monitoring.

#### Critical reflections

Overall, the computational efficiency analysis confirms that the BiGRU–Attention–GWO framework achieves strong predictive performance at moderate resource cost. Accuracy degradation under edge-like constraints was less than 2%, while latency and memory overheads remained feasible for gateway-level devices. However, ultra-constrained IoT nodes may find the memory requirements prohibitive. Future extensions should investigate pruning, quantization, and lightweight neural architectures to further reduce complexity while retaining the accuracy and interpretability of the current design.

### Extended evaluation on additional datasets (CIC-IDS2018 & TON_IoT full)

Table 26 reports the performance of the final optimized HED-ID model on the two newly added datasets using exactly the same hyperparameter configuration obtained via GWO on the original three datasets.

**Table 26:** HED-ID performance on the extended datasets (mean ± std over 10 runs).

**Dataset**	**Accuracy (%)**	**F1-Score**	**AUC**	**Latency (ms)**	**Memory (MB)**
CIC-IDS2018	94.8 ± 0.4	0.94 ± 0.004	0.97 ± 0.002	20 ± 1.2	108 ± 2.5
TON_IoT (full)	93.5 ± 0.5	0.93 ± 0.005	0.96 ± 0.003	21 ± 1.4	112 ± 3.0

The results confirm that HED-ID generalizes effectively to larger and more recent datasets while maintaining the same high accuracy and low resource footprint.

### Conclusion and future work

This study developed the HED-ID Framework, an edge-deployable and explainable intrusion detection system designed to function efficiently across both cloud and edge computing environments. The framework integrates a Stacked BiGRU with Attention, optimized through Grey Wolf Optimization (GWO) and interpreted using SHAP analysis, forming a phased process that spans data preprocessing, optimization, explainable inference, and deployment. Through this design, the framework unifies detection accuracy, interpretability, and computational efficiency within a single methodological structure. Empirical evaluation on the CICIDS-2017, UNSW-NB15, and ToN-IoT datasets yielded accuracies of 95.6% in cloud-like and 93.8% in edge-like settings, with latency between 18–22 ms and memory usage of 92–115 MB. These results demonstrate that the phased architecture of HED-ID functions as the mechanism through which its main contributions—accurate detection, transparent reasoning, and adaptable deployment—are collectively achieved rather than treated as independent objectives.

Certain limitations were observed. Class imbalance continues to affect recall for minority attack categories such as infiltration and heartbleed, while feature overlap occasionally leads to false positives when benign traffic patterns resemble malicious ones. Moreover, the current edge evaluations were conducted in simulated rather than hardware-constrained environments, which limits full assessment of latency and energy performance.

Future work will focus on refining these contributions through targeted extensions. Data balancing and augmentation techniques will be explored to improve detection of minority attacks; context-aware SHAP interpretation will be enhanced to reduce ambiguity in overlapping feature spaces; and hardware-based validation will be performed on real IoT and embedded platforms to assess runtime efficiency. These directions aim to further align the framework’s design with its operational outcomes, ensuring that HED-ID remains a methodologically coherent, explainable, and resource-aware system for practical intrusion detection.

## Supplementary Information

Supplementary Information.

## Data Availability

Underlying data supporting the results are attached in the supplementary material.
